# Protein-centric mechanisms of typhoidal *Salmonella* pathogenicity: emerging opportunities for diagnostics and anti-virulence therapeutics

**DOI:** 10.3389/fcimb.2026.1914114

**Published:** 2026-09-08

**Authors:** Subhash Chandra Pani, Immanuel Dhanasingh

**Affiliations:** Centre for Bio-separation Technology, Vellore Institute of Technology, Vellore, Tamil Nadu, India

**Keywords:** anti-virulence therapeutics, enteric fever, outer membrane proteins, protein-centric pathogenesis, type III secretion system (T3SS), typhoidal *Salmonella*, virulence factors

## Abstract

Typhoidal *Salmonella* (*Salmonella enterica* serovars Typhi and Paratyphi A) continue to impose a significant global health burden, particularly in regions characterized by inadequate sanitation and rising antimicrobial resistance. Although recent reviews have comprehensively addressed epidemiology, antimicrobial resistance, vaccination strategies, and host-pathogen interactions, virulence determinants are often described in a pathway-specific manner. Consequently, a cohesive macromolecular framework integrating these determinants remains insufficiently developed. In this review, we adopt a protein-centric perspective to systematically elucidate the molecular and structural architecture underlying typhoidal *Salmonella* pathogenesis. Diverse virulence determinants, including Type III Secretion System (T3SS) effectors, outer membrane proteins, adhesins, toxin subunits, stress-response regulators, and metabolic proteins, are integrated into a unified structural and functional network in which their three-dimensional architectures, protein–protein interaction interfaces, receptor-binding surfaces, catalytic domains, and active-site configurations collectively govern host-cell invasion, intracellular survival, immune evasion, nutrient acquisition, and systemic dissemination. Furthermore, we present a comparative analysis of *S*. Typhi and *S*. Paratyphi A, emphasizing differences in protein repertoires and regulatory adaptations that drive their distinct pathogenic mechanisms. Collectively, these structural insights establish a mechanistic bridge between protein architecture and pathogenic function and identify molecular interfaces, catalytic pockets, and receptor-binding surfaces as opportunities for diagnostics, vaccines, and anti-virulence therapeutics. Integration of structural biology with artificial intelligence-assisted drug discovery, protein engineering, single-cell infection models, and host-directed approaches may further accelerate the development of next-generation interventions against typhoidal *Salmonella.*

## 1 Introduction

*Salmonella* is a genus of Gram-negative, rod-shaped bacteria responsible for significant foodborne illnesses worldwide. The genus comprises two recognized species, *Salmonella enterica (S. enterica)* and *Salmonella bongori (S. bongori)*. *S. enterica* is further divided into six subspecies and comprises more than 2,500 recognized serovars, the majority of human disease-associated serovars belonging to *S. enterica* subsp. enterica (Andino and Hanning, 2015). Their genomes feature a stable core chromosome (~4.5 Mb) for basic survival and dynamic “pathogenicity islands” acquired via horizontal gene transfer (Sabbagh et al., 2010). Within this species, the human-restricted serovars *S.* Typhi and *S*. Paratyphi A are the principal causes of typhoid and paratyphoid fever, respectively. In contrast, non-typhoidal *Salmonella* serovars are generally associated with gastroenteritis and have broader host ranges. The distinct host restriction and systemic disease phenotype of typhoidal serovars reflect differences in genome content, pathogenicity islands, virulence-associated proteins, and regulatory adaptations (Kuehn et al., 2025; Bharmoria and Behari Vaish, 2016). Enteric fever is common in low- and middle-income countries, with poor sanitation, limited fresh water resources, and overcrowded populations. In 2021, about 9.3 million individuals were affected globally by enteric fever, and around 107,500 deaths were reported. South Asia was the most affected region, contributing to nearly 62% of the total cases all over the world (Piovani et al., 2024). Unlike non-typhoidal *Salmonella* that can infect multiple hosts, such as humans, livestock (poultry, pigs, cattle), and wild animals, as well as household pets (dogs, cats, birds, and reptiles like turtles), typhoidal *Salmonella* exhibits strict human host specificity. *S.* Typhi remains the primary cause of enteric fever, whereas *S.* Paratyphi A has been reported more commonly in recent years (Xie et al., 2022).

The emergence of antibiotic resistance in typhoid treatment represents one of the most serious challenges. In the 1970s, the first outbreak of multidrug-resistant (MDR) typhoid was reported, and was shown to be resistant against chloramphenicol, ampicillin, and cotrimoxazole (Dyson et al., 2019). In 2016, a severe form known as extensively drug-resistant (XDR) typhoidal strain was first discovered in Pakistan. XDR strain is resistant to five classes of antibiotics, including chloramphenicol, ampicillin, cotrimoxazole, streptomycin, fluoroquinolones, and third-generation cephalosporins. As a result, azithromycin became the only effective oral antibiotic available to treat XDR typhoid. However, resistance to azithromycin has recently been reported (Thirumoorthy et al., 2025). When effective antibiotics are no longer responsive against the pathogen, typhoid fever can become life-threatening. Currently, 10-20% of untreated typhoid cases result in death due to complications such as intestinal perforation, hemorrhage, and multi-organ failure (CDC, 2024). Consequently, controlling drug-resistant typhoid fever remains a major challenge in healthcare systems of low- and middle-income countries.

The limitations of antibiotic therapy have driven intense research into alternative therapeutic approaches such as targeted drug therapy, in which specific bacterial proteins, those essential for pathogen survival and infection are targeted for treatment. These proteins govern adhesion to host cells, invasion of the intestinal epithelium, intracellular survival within macrophages, systemic dissemination, and modulation of the host immune response (Li, 2022). The growing limitations of conventional antibiotic therapy against typhoid underscore the urgent need to develop targeted drug therapy against the virulence determinants of typhoidal *Salmonella* (TS). The severity of infection depends on the complex interplay between *Salmonella* proteins and host cell components; a protein-centric approach is, therefore, imperative for the dissection of the molecular details of pathogenesis.

In this review, we integrate structural information with biochemical and functional evidence across virulence associated proteins such as T3SS components, adhesins, outer membrane proteins, LPS and capsule-associated proteins, stress-response regulators, iron-acquisition systems, metabolic enzymes, and typhoid toxin components. Special emphasis is placed on how these structural features translate into virulence phenotypes and create opportunities for diagnostics, vaccine development, and anti-virulence therapeutics.

## 2 Overview of virulence architecture in *Salmonella*

**Figure 1:**
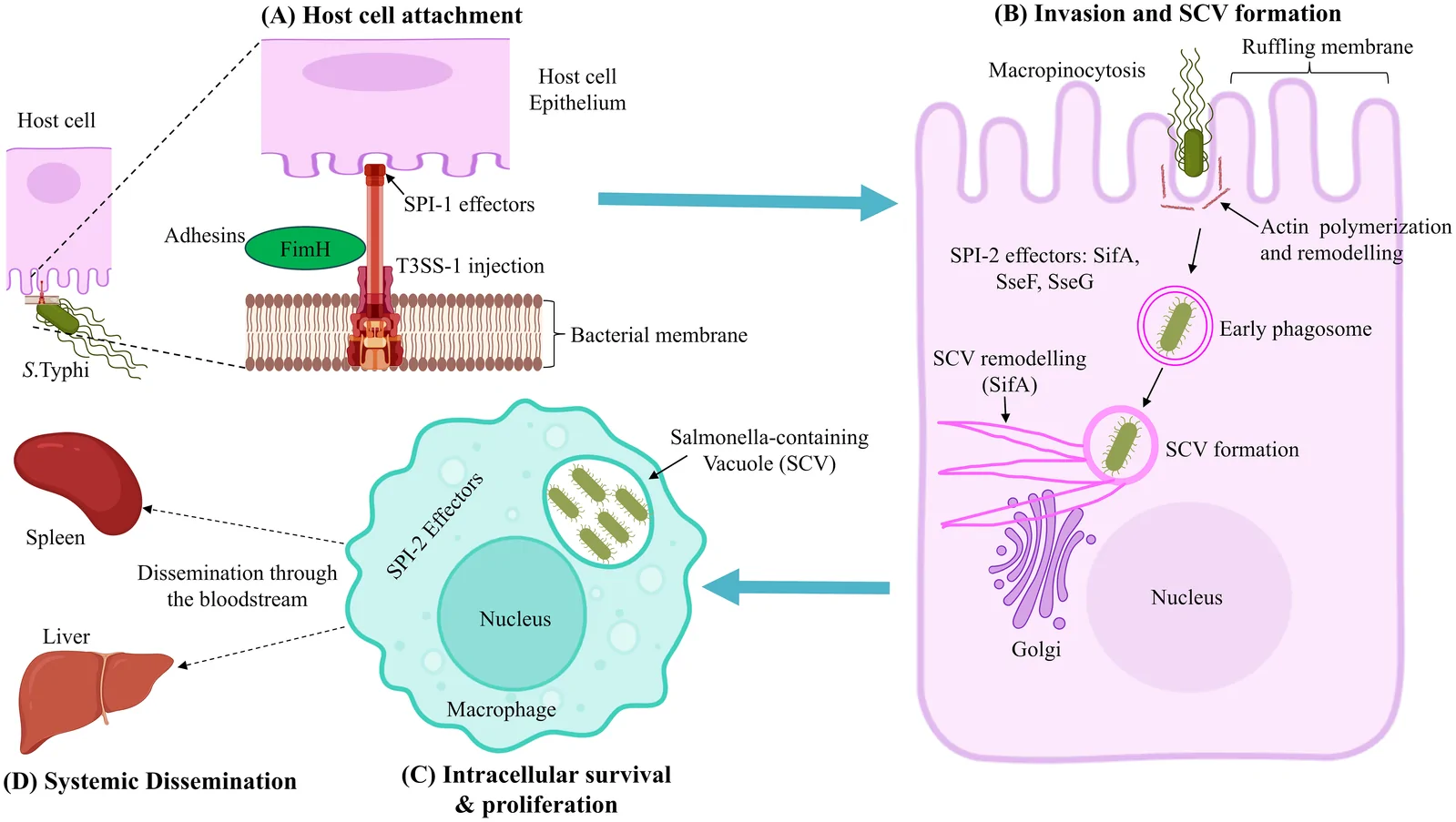
Overview of typhoidal *Salmonella* pathogenicity. **(A)** Initial attachment of *Salmonella* with the host cell epithelium after ingestion through fimbrial adhesins and injection of effector proteins via T3SS. **(B)** Invasion of *Salmonella* via macropinocytosis by SPI-2 effectors and host cytoskeletal rearrangement through actin remodelling, formation of *Salmonella*-containing vacuole and remodelling via sifA. **(C)** Intracellular survival and replication of *Salmonella* inside the macrophage. **(D)** Systemic dissemination of the pathogen through the bloodstream.

The virulence architecture of TS (particularly *S*. Typhi and *S*. Paratyphi A) has a distinct genetic structure that enables human-specific systemic pathogenesis (enteric fever). The pathogenesis begins upon the ingestion of contaminated food, followed by adherence to the host cell, invasion, intracellular survival and infection (**Figure 1**). Following ingestion, bacteria adhere to the intestinal epithelium of the host, where the adhesins and outer membrane proteins mediate attachment to the epithelial cell and microvilli, confirming close contact before invasion (Sima et al., 2024). Next, the invasion process is mediated by a needle-like protrusion called the T3SS, which injects the Spi-1 effectors into the non-phagocytic host cells (**Figure 1A**). These effectors induce membrane ruffling along with actin cytoskeleton rearrangement to facilitate bacterial entry (Lou et al., 2019), (**Figure 1B**). Upon bacterial invasion, the bacteria are enclosed in a membrane- bound compartment called a *Salmonella*-containing vacuole (SCV), formed by SPI-1 effectors (Malik-Kale et al., 2011), (**Figure 1C**). Furthermore, the SPI-2 effectors maintain the integrity of SCV, prevent lysosomal fusion and facilitate intracellular survival and replication. Hereafter, the pathogen survives inside the macrophages for a longer time period, where it produces additional toxin components and travels via the bloodstream to the liver, spleen, bone marrow, causing systemic infection (**Figure 1D**) (Zhao et al., 2025).

This infection is further initiated by the synergistic action of highly conserved virulence factors such as *Salmonella* Pathogenicity Islands (SPIs), adhesins, outer membrane proteins (OMPS), stress regulators, iron regulators and toxin components. From bacterial entry through the fecal-oral route to its survival and dissemination within macrophages, the following effectors play a crucial role.

### 2.1 *Salmonella* pathogenicity islands

**Figure 2:**
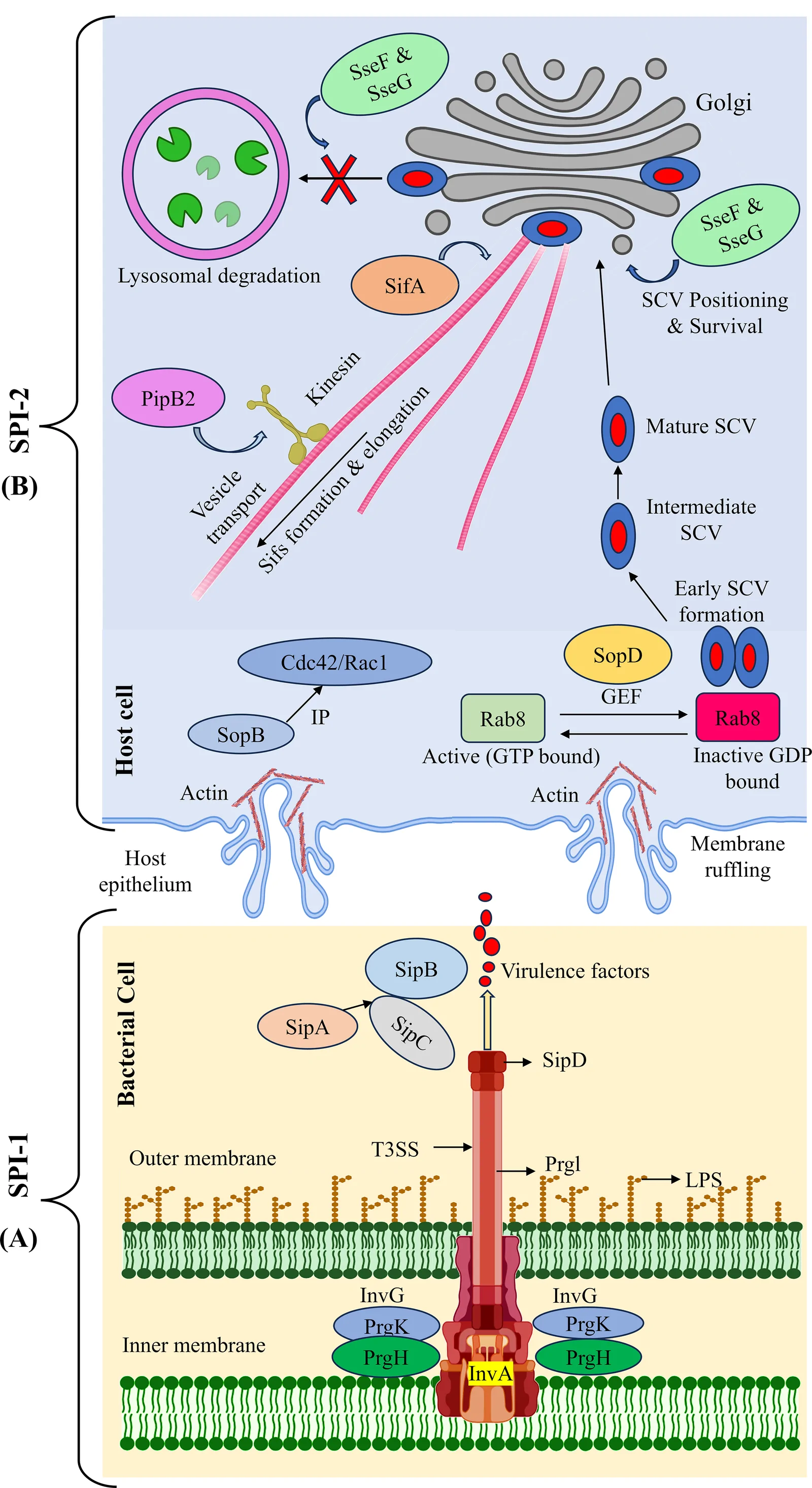
Host–pathogen interactions mediated by the coordinated activities of SPI-1 and SPI-2 effector proteins in *Salmonella*. **(A)** During bacterial attachment to the host epithelium, the *Salmonella* Type III Secretion System (T3SS) encoded by *Salmonella* Pathogenicity Island 1 (SPI-1) injects effector proteins directly into host cells. These effectors induce actin cytoskeleton remodeling, membrane ruffling, and bacterial internalization, thereby facilitating invasion of the host epithelium. **(B)** Following invasion, *Salmonella* resides within a membrane-bound compartment known as the *Salmonella*-containing vacuole (SCV). Effector proteins delivered by the SPI-2-encoded T3SS promote SCV biogenesis, intracellular survival, and replication. Additional SPI-2 effectors regulate SCV positioning and trafficking within the host cell while preventing lysosomal fusion and degradation, thereby enabling persistent intracellular infection.

*Salmonella* pathogenicity islands (SPIs) are extensive clusters of genes located within the chromosome, acquired through horizontal gene transfer from other organisms. These islands encompass critical virulence factors that facilitate the invasion, survival, and pathogenicity of *Salmonella* within host cells (**Table 1**). Major islands include SPI-1 which facilitates the invasion of non-phagocytic intestinal epithelial cells by delivering effector proteins via a T3SS, resulting in membrane ruffling and macropinocytosis (**Figures 1A**, **2**), (Wang et al., 2020; Lou et al., 2019). SPI-2 ensures intracellular survival and systemic dissemination by maintaining the SCV within macrophages (**Figures 1B, C**, **2**). The SCV prevents phagolysosomal fusion and modulates immunological responses when engulfed by host macrophage (**Figure 1C**), (Wang et al., 2020). SPI-3 supports bacterial adaptation under nutrient-limited conditions, particularly low magnesium environments, and SPI-4 promotes adhesion to host cells for effective colonization. SPI-5 contributes to effector translocation and modulation of host immune responses. *S.* Typhi’s systemic pathogenicity is primarily associated with SPI-7, which includes the viaB locus encoding the Vi capsular polysaccharide that inhibits complement deposition, lowers phagocytosis, and masks LPS from TLR-4 recognition (Lou et al., 2019; Zhao et al., 2025). Vi capsular regulator, TviA, lowers flagellin and T3SS-1 expression, thereby reducing TLR-5 and NAIP-mediated inflammation (Winter et al., 2015). Despite lacking the Vi capsule, *S.* Paratyphi A expresses fepE, which promotes very long O-antigen chains that suppress inflammasome activation and host cell death (Mylona et al., 2021). SPI-9 encodes an RTX-like (Repeat in toxin) protein and a type I secretion system (Parkhill et al., 2001), while SPI-10 carries the self-fimbrial operon, potentially contributing to host specificity (**Table 1**) (Townsend et al., 2001). In typhoidal serovars, genes encoding typhoid toxin components (cdtB, pltA, and pltB) contribute to systemic pathogenesis through host cell intoxication (Spanò et al., 2008; Srour et al., 2026). SPI-16 and SPI-17 encode GtrA and GtrB translocases, which are involved in O-antigen glycosylation and serovar conversion (Vernikos and Parkhill, 2006), and SPI-18, present in *S.* Typhi but not in *S.* Typhimurium, contains hlyE, which encodes a pore-forming hemolysin (Fuentes et al., 2008).

**Table 1:** *Salmonella* pathogenicity islands and associated protein functions.

SPI	Major protein components	Primary function	Relevance in typhoidal *Salmonella*
SPI-1	SipA, SipC, SopB, SopD, InvA	Actin remodeling and epithelial invasion via T3SS-1	Essential for host cell entry and initiation of infection (Wang et al., 2020; Lou et al., 2019)
SPI-2	SifA, SseF, SseG, PipB2, SpiC	Intracellular survival and SCV maintenance via T3SS-2	Critical for macrophage survival and systemic dissemination (Wang et al., 2020)
SPI-3	MgtC, MisL	Magnesium uptake and intracellular adaptation	Supports survival under nutrient-limited conditions (S et al., 2019)
SPI-4	SiiE, SiiF	Adhesion and epithelial interaction	Contributes to host cell attachment and colonization (C et al., 2014)
SPI-5	SopB, PipB	Effector translocation and inflammation modulation	Enhances intracellular trafficking and immune modulation (Szeto et al., 2009)
SPI-7 (*S.* Typhi)	TviA, TviB, TviC, TviD, TviE	Vi capsule biosynthesis and immune evasion	Unique determinant of *S.* Typhi pathogenicity (Wang et al., 2023), (Wang et al., 2020; Gal-Mor et al., 2014).
SPI- 9	BapA, membrane fusion proteins	Adhesion and host colonization, biofilm formation	Contributing to stable host surface attachment (Parkhill et al., 2001)
SPI- 10	SeF, PrpZ	Important for bacterial survival and proliferation	Host specificity in *S.*Typhi (Townsend et al., 2001)
SPI-11	CdtB, PltAB	DNA damage and cell cycle arrest	Unique typhoid toxin for typhoidal *Salmonella* (Spanò et al., 2008; Srour et al., 2026)
SPI-16 & 17	GtrA and GtrB	O-antigen glycosylation and serovar conversion	Increases resistance to complement-mediated killing (Vernikos and Parkhill, 2006)
SPI-18 (*S.* Typhi)	HlyE	Pore- forming hemolysin	Mediates hemolysis in the host cell (Fuentes et al., 2008)

Together, these SPIs orchestrate invasion, survival, immune evasion, and pathogenicity in typhoidal *Salmonella*.

### 2.2 Virulence of typhoidal *vs* non-typhoidal *Salmonella*

Enteric fever caused by typhoidal serovars differs dramatically from the gastroenteritis normally associated with non-typhoidal *Salmonella* (NTS) (**Table 2**). The average incubation period for typhoidal serovars is 14 days, with symptoms persisting for up to 3 weeks (Wangdi et al., 2012). With NTS infection, symptoms appear 6–12 h after the ingestion of the pathogen and clinical symptoms last less than 10 days (Glynn and Palmer, 1992). Both typhoidal and NTS serovars initially adhere to and invade the intestinal epithelium of the small intestine. Unlike NTS infection, infection by typhoidal serovars does not induce a high inflammatory response during the initial invasion of the intestinal mucosa (Nguyen et al., 2004). Notably, typhoidal *Salmonella* have undergone genome degradation, accumulating pseudogenes (inactive genes) compared to non-typhoidal *Salmonella*, which are required for survival in diverse hosts. This is the reason why typhoidal *Salmonella* becomes highly specific to the human host (Hiyoshi et al., 2018). In immunocompetent patients, NTS gastroenteritis is self-limiting, with infection being confined to the terminal ileum and colon. In the case of typhoidal *Salmonella*e, after passing the intestinal mucosa, bacteria gain access to underlying lymphoid tissues and multiply intracellularly within phagocytes (Gordon, 2008). These specific features cause NTS to be more acute and less pathogenic in human compared to TS that is chronic and pathogenic.

**Table 2:** Distinctive features of typhoidal and non-typhoidal *Salmonella*.

Feature	Typhoidal *Salmonella*	Non-typhoidal *Salmonella*
Host range	Human-restricted	Broad (Wang et al., 2020; Buzilă et al., 2025; Johnson et al., 2018b; Gal-Mor et al., 2014; J Barton et al., 2021)
Key Serovars	S. Typhi, Paratyphi A, B, C	*S.* Typhimurium, Enteritidis, Dublin (Wang et al., 2020; Buzilă et al., 2025)
Unique Toxin	A_2_B_5_ typhoid toxin	Virulence is primarily mediated by T3SS and *Salmonella* Pathogenicity Islands (SPIs) (Wang et al., 2020; Johnson et al., 2018b; Gal-Mor et al., 2014)
Antigen	Vi antigen.	NA (Wang et al., 2020; Gal-Mor et al., 2014; Johnson et al., 2018b)
Flagella/T3SS-1	Downregulated by regulatory elements (e.g., TviA).	Highly Expressed (Wang et al., 2020)
Inflammatory Response	minimal/weak	Robust, acute inflammatory response (gastroenteritis) (Wang et al., 2020; J Barton et al., 2021).
Clinical menifestation	Invasive, systemic disease in immunocompetent individuals	Self-limiting gastroenteritis in immunocompetent individuals (Johnson et al., 2018b; J Barton et al., 2021).
Vaccination	live attenuated oral vaccine (Ty21a), Vi polysaccharide capsule-based vaccine	No vaccine available for humans rather than poultry (Gal-Mor et al., 2014)

In case of vaccines, there are two types of vaccines (Ty21 and Vi capsular) currently available for typhoidal *Salmonella* caused by *S.* Typhi. However in NTS, there are vaccines available only for poultry (Gal-Mor et al., 2014; **Table 2**).

## 3 Protein families central to pathogenesis

### 3.1 Type III secretion system: coordinated effector functions in infection

The T3SS is a central virulence apparatus that enables *Salmonella* to directly manipulate host cellular processes by delivering effector proteins into the host cell (**Table 3**). Two functionally distinct systems, encoded by SPI-1 and SPI-2, operate sequentially but in a coordinated manner to establish infection (Glynn and Palmer, 1992; Nguyen et al., 2004) (**Figure 2**).

**Table 3:** Key protein determinants of typhoidal *Salmonella* pathogenesis.

Protein class	Representative proteins	Functional role	Translational potential
T3SS effectors	SipA, SipC, SopB, SifA	Host invasion and intracellular survival	Targets for anti-virulence inhibitors (Cain et al., 2008)
Adhesins	FimH, T2544, StkF	Host cell attachment and colonization	Vaccine candidates (Wagner and Hensel, 2011; Kolenda et al., 2019; Uchiya et al., 2019; Wizemann et al., 1999; Ghosh et al., 2011)
Outer membrane proteins	OmpA, OmpC, OmpF, LamB	Membrane stability and immune interaction	Broad-spectrum vaccine antigens (Belousov et al., 2023; Nevermann et al., 2019)
Stress response proteins	DnaK, GroEL, HtrA	Survival under oxidative and thermal stress	Biomarkers and attenuation targets (Tang et al., 1997; Zhu et al., 2015)
LPS/capsule proteins	LpxA, LpxC, Tvi proteins	Immune evasion and membrane integrity	Drug targets and diagnostic markers (Zhou and Zhao, 2017; Hummels, 2025; Raetz et al., 2007), (H. Lee et al., 2004)
Iron acquisition systems	Enterobactin, Iro proteins	Iron scavenging under host restriction	Targets for metabolic inhibition (Wellawa et al., 2022; Fernandez-Beros et al., 1989; Nagy et al., 2013; Jia et al., 2022; Hantke et al., 2003; Crouch et al., 2008; Costa et al., 2016)
Toxin components	CdtB, PltA, PltB	Host DNA damage and systemic toxicity	Targets for neutralizing antibodies (Chang et al., 2022; Song et al., 2013; Fowler et al., 2019)

SPI-1 is primarily activated during the extracellular phase and facilitates bacterial entry into non-phagocytic epithelial cells (**Figure 2A**). This is achieved through the concerted action of effectors that remodel the host actin cytoskeleton and plasma membrane (Ramos-Morales, 2012). Proteins such as SipA, SipB and SipC promote actin polymerization and stabilization, CryoEM structure of SipA revealed the actin-binding architecture that stabilizes filaments and promotes polymerization. The C-terminal globular domain (residues ~500-657) creates four distinct interfaces with actin subunits N, N + 1, N + 2, N + 3 (PDB: 8VFM). This is unusual compared to other actin-binding proteins, which typically interact with one or two subunits. Furthermore, it creates hydrophobic cores that stabilize the geometry (e.g., SipA K636 with actin C-terminus, SipA S537 with actin D-loop). The C-terminal extension region (residues 658–675) is a flexible tail that acts like a polymerization hook, which is Essential for G-actin nucleation/polymerization, but not required for F-actin stabilization (Guo et al., 2023). SipB is a key virulence translocon protein that work with SipC and SipD to form a pore complex in the host cell membrane. The crystal structure of N-terminal SipB (residues 82–226) (PDB: 3TUL) reveals a long intramolecular coiled-coil motif composed of three antiparallel α-helices. The hydrophobic C-terminal domain is predicted to form a transmembrane hairpin that anchors SipB into host membranes. It oligomerizes at the needle tip, creating a conduit for effector proteins (Barta et al., 2012). SipC is a ~43 kDa protein with an N-terminal (1–120 aa) with signal peptide and chaperone-binding motif, a central hydrophobic core (~80 aa) and a C-terminal region (~321–409 aa), critical for effector translocation, actin modulation, and SipB binding. It can self-associate into dimers, trimers, and higher-order oligomers, which is essential for pore formation in host membranes (Myeni et al., 2013). SipD is the tip protein which are majorly α- helices (~62% residues in helices). It contains an N-terminal region with helices α1 and α2 forming an α-helical hairpin that packs against the coiled coil; helix α3 and a flexible loop (residues 110–132) flank the opposite side (PDB: 3NZZ). SipD likely forms a multimeric complex (pentamer/hexamer) at the needle tip, consistent with other T3SS tip proteins leading to membrane ruffling and bacterial uptake. Furthermore, SipD has one bile salt binding pocket in the active site, which involves hydrophobic interactions with residues such as F340, L318, L322, and I45, and hydrogen bond interactions with residues such as K338 and N321 (Chatterjee et al., 2011). SopB (also known as SigD) plays a sophisticated and dual-faceted role in modulating the host small GTPases Cdc42 and Rac1 during *Salmonella* invasion (Burkinshaw et al., 2012). Structurally, It possesses a specialized N-terminal Cdc42-binding domain (PDB: 4DID) that targets Cdc42 directly through structural mimicry and influences Rac1 indirectly through its lipid phosphatase activity, while related *Salmonella* effectors like SopE and SopE2 act as direct Guanine Nucleotide Exchange Factors (GEFs) to rapidly activate host Rho GTPases, especially Cdc42 and Rac1, to amplify cytoskeletal rearrangements (Piscatelli et al., 2016). (**Figure 2**; **Table 3**). Structurally, SopE consists of six α-helices arranged into two three-helix bundles, forming a V-shaped architecture. The key interactions involved the formation of salt bridge (Asp38 (Cdc42) ↔ Lys198 (SopE)) and hydrogen bond interaction (Tyr40 (Cdc42) ↔ Gln194 (SopE)) (PDB: 1GZS) (Buchwald et al., 2002). SopD play a regulatory role in membrane trafficking through Rab8 recruitment to the SCV (**Figure 2**). These events culminate in the formation of the *Salmonella*-containing vacuole (SCV), which serves as the intracellular niche (**Figure 2**) (Friebel et al., 2001; Fass and Groisman, 2009; Johnson et al., 2018a).

Following internalization, SPI-2 becomes dominant and supports intracellular survival and replication (**Figure 2B**). Effectors such as SifA, SseF, and SseG regulate SCV positioning and integrity, ensuring avoidance of lysosomal degradation and enabling interaction with host trafficking pathways. SifA is not a catalytic enzyme but a virulence effector with a two-domain architecture. Its active site is the N-terminal SKIP-binding interface, stabilized by the WXXXE motif in the C-terminal domain (PDB: 3CXB). The protein functions as a molecular scaffold rather than a catalyst, mediating host-pathogen interactions essential for *Salmonella* survival (Diacovich et al., 2009). SseF contains a functionally important C-terminal hydrophobic region with three predicted transmembrane helices that facilitates its association with SCV/endosomal membranes and is required for juxtanuclear, Golgi-associated positioning of the SCV (Abrahams et al., 2006). SseF also interacts functionally and physically with SseG, which associates with endosomal membranes and microtubules. Together, SseF and SseG promote microtubule-dependent positioning of the SCV and recruitment of host trafficking components, while SifA coordinates membrane dynamics with kinesin-dependent transport (Abrahams et al., 2006). PipB2 further contributes by recruiting motor proteins (kinesins) that facilitate intracellular movement of the SCV (**Figure 2**; **Table 3**).

**Figure 3:**
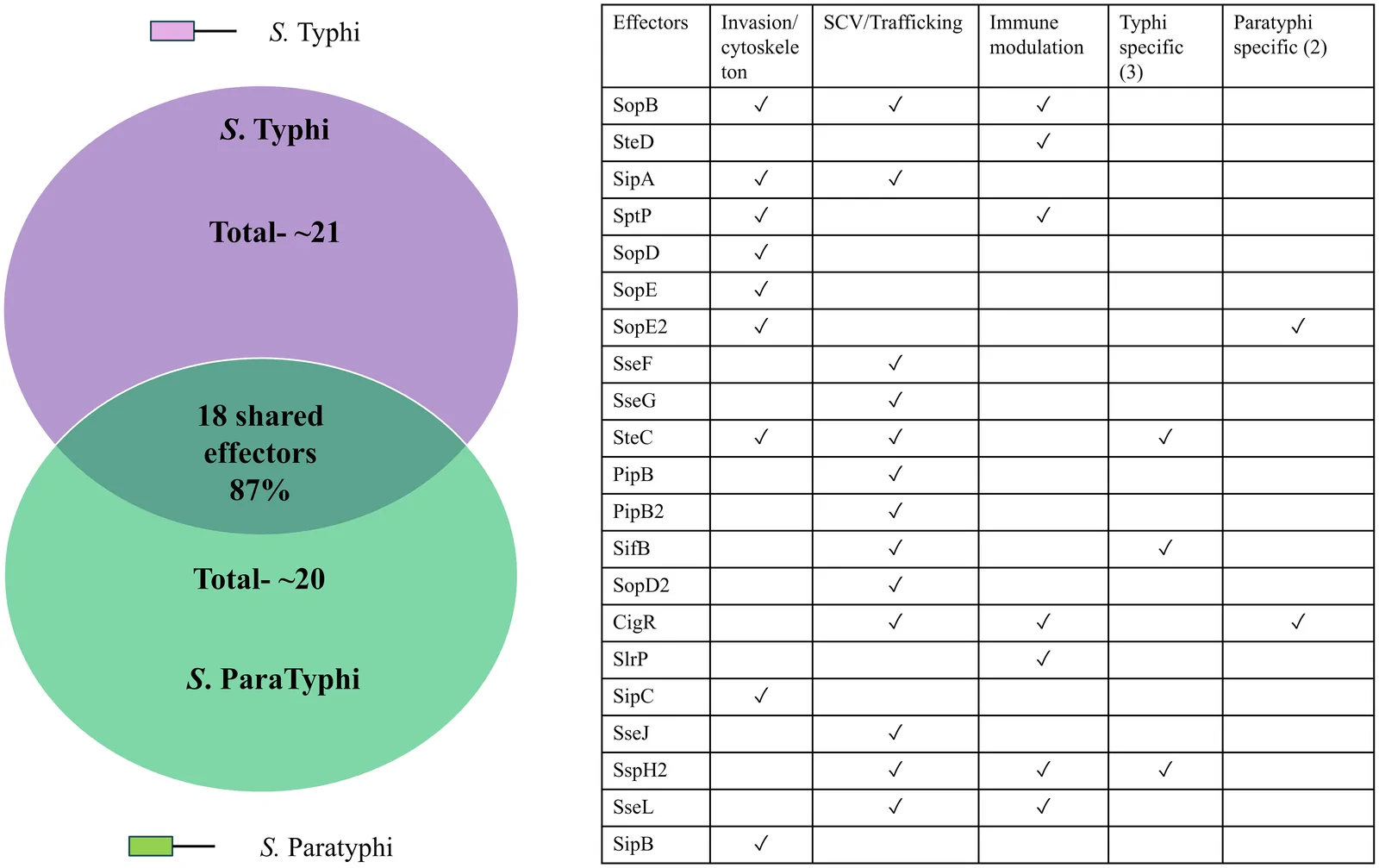
An illustration of number of effectors of T3SS in *S*. Typhi and *S*. Paratyphi A. The Venn diagram summarizes the shared and serovar-specific effector repertoires, with 18 shared effectors, three *S*. Typhi-specific effectors (SteC, SifB and SspH2), and two S. Paratyphi A-specific effectors (SopE2 and CigR). The accompanying classification summarizes the distribution of these effectors according to their major functional roles in host-cell invasion, cytoskeletal remodeling, intracellular trafficking and immune modulation. The comparison is based on the reported effector repertoires of *S*. Typhi Ty2 (CT18) and *S*. Paratyphi A ATCC 9150.

Although PipB2 lacks a conventional active site, its protein–protein interaction surfaces could potentially be targeted. In particular, disrupting the PipB2–KLC interaction could interfere with recruitment of kinesin-1 to the SCV and consequently alter *Salmonella* intracellular trafficking. Experimental studies demonstrate that PipB2 translocation is sufficient to recruit kinesin-1 to the SCV membrane (Henry et al., 2006). Typhoidal serovars exhibit selective retention and loss of effectors as compared to non-typhoidal strains, reflecting adaptation to a systemic infection strategy. More than 40 T3SS effectors have been identified in *S*. Typhimurium so far. From these, approximately 21 effectors are either absent or present as pseudogenes in *S.* Typhi. From the active 21 effectors, 18 effectors are shared with *S*. Paratyphi A, while 3 effectors (SteC, SifB, and SspH2) are unique to *S*. Typhi. Similarly, *S. Paratyphi* A possesses 20 T3SS effectors, of which two, SopE2 and CigR, are unique to this serovar (**Figure 3**) (Johnson et al., 2018a; Ramos-Morales, 2012). This streamlined effector repertoire suggests that typhoidal *Salmonella* relies on a refined subset of proteins optimized for immune evasion and persistence within human hosts.

Collectively, SPI-1 and SPI-2 function as a coordinated invasion-survival axis (**Figures 2**, **3**), where early cytoskeletal manipulation is seamlessly linked to intracellular adaptation, forming a critical foundation for systemic disease progression.

### 3.2 Adhesins and outer membrane structures

#### 3.2.1 Adhesins

**Figure 4:**
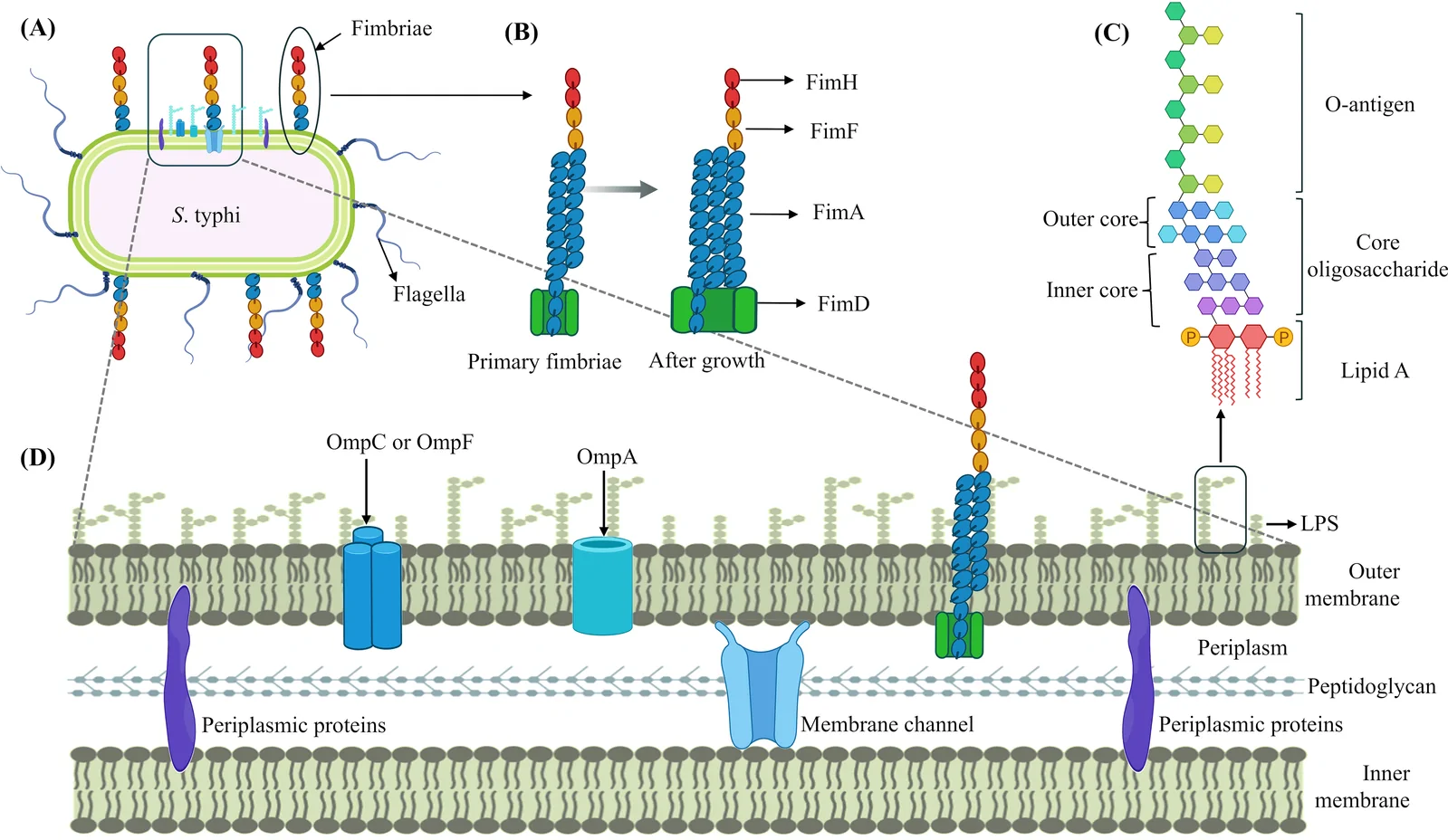
Fimbrial adhesins and lipopolysaccharide (LPS) structures involved in *Salmonella* host colonization and virulence. **(A)** Schematic representation of the *Salmonella* cell surface showing fimbrial adhesins and lipopolysaccharides (LPS), two major surface structures involved in host-pathogen interactions. **(B)** Fimbrial adhesins extending from the bacterial surface mediate attachment to host epithelial cells, facilitating colonization and the establishment of infection. **(C)** Structural organization of *Salmonella* lipopolysaccharide, consisting of lipid A, the core oligosaccharide, and the O-antigen polysaccharide. Lipid A anchors the LPS to the outer membrane and contributes to endotoxic activity, whereas the O-antigen plays a key role in immune evasion, serum resistance, and bacterial virulence. **(D)** Schematic representation of the *Salmonella* cell envelope showing the outer membrane contains major porins (OmpC and OmpF) involved in the diffusion of small molecules, as well as OmpA, which contributes to membrane integrity and host interactions. Surface-associated fimbriae (pili) extend from the outer membrane and facilitate adhesion to host tissues and biofilm formation.

Host cell adhesion represents a critical early step in bacterial colonization and infection (Kline et al., 2009). During attachment (**Figure 1**), bacterial surface adhesins recognise and bind host cell surface receptors, such as glycosaminoglycans (GAGs), and in other cases they bind with the extracellular matrix (ECM) components, such as collagen, laminin, fibronectin, heparan sulfate, and polyhydroxylated glycan (Rehman et al., 2019). Fimbriae, a type of adhesin, are extracellular appendages with 0.5-10 μm length and 2-8 nm width, are typically involved in bacterial adhesion to the host (**Figure 4A**), in addition to many other functions such as interaction with macrophages, biofilm formation, intestinal persistence, and bacterial aggregation (Duguid et al., 1955; Althouse et al., 2003). The base of the fimbriae is attached to the bottom end of the outer membrane while the tip of the fimbriae is exposed to the surface (**Figure 4B**). Four subunits of fimbriae (such as FimH, FimF, FimA and FimD) assemble to make mature fimbriae (**Figure 4B**). FimD is a β-barrel outer membrane protein that acts as the anchoring subunit attaches to the surface, after which the other subunits, FimA, FimF, and FimH, are assembled on top (**Figure 4B**). The initial primary fimbriae lacks specific adhesion to the host due to the smooth bacterial surface; after growth and maturation, it gets a filamentous surface, which has stronger and specific binding to host cells (**Figure 4B**) (Kolenda et al., 2019; Uchiya et al., 2019). The type 1 fimbrial adhesin, FimH subunit, the tip fimbriae, has been intensively characterized structurally and functionally (Wagner and Hensel, 2011). Structurally, FimH comprises an N-terminal lectin domain containing a conserved mannose-binding pocket and a C-terminal pilin domain that anchors the adhesin to the fimbrial shaft (PDB: 9AT9). Binding of the lectin domain to mannosylated glycoproteins on epithelial cells mediates high-affinity, mannose-sensitive adhesion, while interaction with Toll-like receptor 4 (TLR4) activates macrophage inflammatory responses **Figure 4**) (Wizemann et al., 1999; Lopatto et al., 2024).

Beyond adhesion, FimH is a promising target for diagnostics and intervention. Its conserved and surface-exposed nature makes it an effective vaccine antigen, with recombinant and mRNA-based vaccines eliciting robust antibody and T-cell responses in preclinical studies. In addition, FimH-directed anti-adhesion therapeutics, particularly mannoside inhibitors, effectively inhibit bacterial attachment to host cells, highlighting its translational potential (Roier et al., 2025). A recently discovered mannose-based nanozyme biosensor (Man-MPN) utilized the particular FimH-mannose interaction in order to capture *S.* Typhimurium, enabling quick and sensitive detection. This preclinical analytical platform provided a visual colorimetric readout within approximately 15 min, without requiring antibodies or labelled probes, and achieved a detection limit of approximately 10³ CFU/mL, highlighting the potential of FimH-mediated recognition for rapid and accessible *Salmonella* diagnostics (Deng et al., 2026).

#### 3.2.2 Outer membrane proteins

Outer membrane proteins (OMPs) of Gram-negative bacteria, such as *S.* Typhi, represent porins that are involved in critical functions such as transport across membranes, adhesion to the host, penetration, and colonization, which lead to virulence and pathogenesis in host tissues (Belousov et al., 2023) (**Figure 4D**). During direct bacterial contact with the host, membrane protein OmpA contributes to bacterial survival within host cells by enhancing nitric oxide-mediated stress resistance, promoting intracellular replication, and modulating vesicle-mediated toxin secretion (Nevermann et al., 2019). OmpC and OmpF are trimeric β-barrel porins that provide support for nutrient permeability and membrane homeostasis, and are involved in additional host-interactive properties through inflammation-linked amyloid-like aggregation (**Figure 4D**) (Belousov et al., 2023). OmpC contains surface-exposed extracellular loops that are accessible to antibodies. Studies mapping OmpC epitopes demonstrated that some monoclonal antibodies recognize exposed loops, whereas other antibodies recognize conserved, buried β-strand regions (Singh et al., 1995).

Porins such as OmpC and OmpF are strong immunogens and can naturally enhance immune responses due to their intrinsic adjuvant-like properties. When used together, they broaden B-cell responses and promote effective antibody class switching, making them promising candidates for subunit vaccines or inclusion in multivalent vaccine formulations (Pérez-Toledo et al., 2017). TolC contributes to antimicrobial resistance and virulence by mediating efflux and regulating virulence gene expression. Studies found that its deletion showed a significant reduction in adhesion and invasion towards host cells, making it a promising target for vaccine development and novel antimicrobial therapies (Hussain et al., 2023). A recent study in 2024 showed that, in the absence of TolC, the immune system, especially macrophages, responds more aggressively to the bacterium (Hussain et al., 2024). The above *in vitro* preclinical studies confirm that TolC is a major regulatory factor of bacterial invasion and intracellular survival within host cells.

#### 3.2.3 Biofilm formation proteins

Biofilm-associated protein (Bap) has a major role in the production of biofilm composed of cellulose and curli fimbriae. Curli fibers are amyloid proteins expressed by enteric bacteria, including *E. coli* and *S. enterica*, play a pivotal structural component of the biofilm ECM (Adcox et al., 2016). Curli genes are arranged in two divergent curli-specific gene (*csg*) operons *csgBAC* and *csgDEFG* with independent promoters: one contains the structural components CsgA and CsgB (*csgBAC* operon) and the other contains the regulator CsgD and other structural and facilitator proteins (*csgDEFG* operon) (González et al., 2024).

Structurally, CsgA is composed of five repeating motifs (R1–R5), each contributing β-strands that stack into a β-solenoid fold. In solution, CsgA is largely disordered, but upon nucleation by CsgB, it adopts a highly ordered cross-β amyloid structure. This arrangement gives curli fibers their remarkable stability and resistance to proteolysis (Bu et al., 2024). CsgD is the master transcriptional regulator in *E. coli* and related enterobacteria, central to biofilm formation and curli fiber production. Unlike CsgA, which is a structural amyloid protein, CsgD is a DNA-binding protein that controls the expression of multiple genes involved in extracellular matrix assembly and stress adaptation. Structurally, CsgD belongs to the LuxR/UhpA family of transcriptional regulators. It has two distinct domains: an N-terminal receiver domain that resembles response regulators in two-component systems, and a C-terminal helix-turn-helix (HTH) DNA-binding domain (PDB: 5XP0). The N-terminal domain is thought to mediate dimerization and regulatory input, while the C-terminal domain directly interacts with promoter DNA sequences to activate transcription. Crystallographic studies and homology modeling suggest that CsgD forms dimers, which is essential for stable DNA binding and transcriptional activation (Wen et al., 2017). Expression of Bap is positively regulated by CsgD (Latasa et al., 2005). These proteins enable the bacteria to produce biofilms that facilitate survival under host immune pressure, host colonization, increases antimicrobial tolerance and finally promotes survival under host cell environment (Uruén et al., 2020).

Biofilm regulators such as AdrA, BcsA, and CsgD are essential for forming and controlling the biofilm matrix, which allows bacteria to thrive in adverse conditions. Targeting CsgD to prevent biofilm formation, or employing enzymes such as cellulase to degrade extracellular cellulose within the biofilm, are potential strategies for eradicating chronic carriers and treating long-term infections in the biliary system or gall bladder (Gunn et al., 2016; Upadhyay et al., 2024).

### 3.3 LPS and capsule biosynthesis

Despite Lipopolysaccharide (LPS) being a glycolipid and not a protein, is a major outer membrane component of *S*. Typhi and acts as a critical virulence factor, structural barrier, and primary target for the host’s immune system. LPS are composed of a hydrophilic heteropolysaccharide which contains three key structural domains: Lipid A, Core oligosaccharide and O-antigen (**Figure 4C**) (Hoare et al., 2006). The primary role of Lipid A is to anchor LPS in the bacterial membrane and is the component responsible for triggering toxic shock in severe infections. The Core oligosaccharide connects lipid A to the O-antigen and is critical for the early stages of epithelial cell adherence and invasion. Finally, the O-Antigen is the surface-exposed polysaccharide chain that protects the bacteria from host immune defences and complement-mediated killing (Caroff and Novikov, 2020). Although LPS is a common structural component of all *Salmonella* serovars, *S*. Typhi possesses an additional capsular polysaccharide known as the Vi antigen that surrounds the bacterial surface and aids the pathogen in evading host immune responses. In contrast, *Salmonella* Paratyphi lacks the Vi antigen but expresses a specialized elongated O-antigen as part of its LPS structure, which contributes to immune evasion by masking surface components, thereby enhancing bacterial survival within the host (Guerra et al., 2025).

#### 3.3.1 Lipid A biosynthesis

Owing to Lipid A’s importance in pathogenicity, the Raetz pathway of lipid A biosynthesis has been the primary therapeutic target in Gram negative bacterial infections. This pathway involves nine enzymes in *E. coli*, of these, six (LpxA, LpxC, LpxD, LpxH/I/G, LpxB, LpxK) are essential and prime antibiotic targets, whereas three (KdtA, LpxL, LpxM) are non-essential and contribute to virulence (Zhou and Zhao, 2017; Hummels, 2025; Raetz et al., 2007). Among these, LpxC is a highly conserved Zn²^+^-dependent metalloamidase that catalyzes the committed deacetylation step in lipid A biosynthesis. Structurally, it comprises two α/β domains that form a deep catalytic cleft, where the Zn²^+^ ion is coordinated by conserved H79-H238-D242 residues to activate a water molecule for substrate hydrolysis (PDB: 1P42) (Whittington et al., 2003) Additionally, it contains a hydrophobic tunnel for acyl chain binding which formed by residues like Leu18, Ile102, Thr75 and a sugar binding pocket with the polar residues such as Lys238 and His264 that interact with the ribose and glucosamine moieties of the substrate (Furuya et al., 2020).This well-defined metal-binding active site has made LpxC a prominent target for structure-guided inhibitor development (PDB: 5VWM), including hydroxamate-based compounds such as CHIR-090 (Cole et al., 2011). The first-ever LpxC inhibitor (ACHN-975) against *Pseudomonas aeruginosa* was reported in 2019, and this is the only inhibitor that reached human clinical trials; however, the clinical trial was terminated due to side effects (hypertension) (Krause et al., 2019). Another LpxC inhibitor, RC-01, which was developed against Gram-negative bacteria such as *Pseudomonas aeruginosa*, *Acinetobacter baumannii* and *Enterobacteriaceae*, was also discontinued from the phase-1 clinical trial for safety reasons (Recida Therapeutics, Inc., 2019). LpxH is a membrane-associated metalloenzyme that hydrolyzes UDP-2,3-diacylglucosamine to generate lipid X, a key intermediate in lipid A biosynthesis (Karthikeyan et al., 2025; Young et al., 2013). Structurally, its catalytic pocket contains a conserved Mn²^+^-dependent active site that coordinates substrate hydrolysis through a metal-assisted mechanism, making it highly amenable to structure-based inhibitor design (Kwak et al., 2023). Structure-guided optimization of pyridinyl sulfonyl piperazine inhibitors led to the development of JH-LPH-106 (PDB: 9CD0) and JH-LPH-107 (PDB: 9CD1), which exhibited potent antibacterial activity against wild-type Enterobacterales, including *Klebsiella pneumoniae* and *Escherichia coli*. Among these compounds, JH-LPH-107 emerged particularly promising lead, with a low potential for spontaneous resistance and an excellent *in vitro* safety profile (Ennis et al., 2024). LpxA is a trimeric acyltransferase with a left-handed β-helix core and a small C-terminal α-helical domain. Its active site lies at the monomer interface, where His121 acts as the catalytic base and Gly139 helps stabilize the transition state (PDB: 5DEM). Substrate binding involves residues like Asn194, Arg201, His156, and Tyr158, while Met169 serves as the “hydrocarbon ruler” that enforces preference for C10 acyl chains (Smith et al., 2015). The crystal structure of *E. coli* LpxD (EcLpxD) revealed a homotrimeric enzyme in which each monomer comprises three distinct structural regions: an N-terminal uridine-binding domain, a central left-handed parallel β-helix (LβH) domain, and a C-terminal α-helical domain (PDB: 3EH0). The LβH region forms the main catalytic core and contains conserved residues for substrate binding and catalysis. The N-terminal domain facilitates the binding of the UDP-containing substrate. An adjacent hydrophobic cleft accommodates the acyl chain of the R-3-hydroxyacyl-ACP donor substrate. Notably, Met290 (M290) is positioned at the distal end of this hydrophobic cleft and contributes to determining the acyl-chain specificity of LpxD (Bartling and Raetz, 2009).

RJPXD33 is a dual-target inhibitory peptide that mimics the β-hydroxyacyl substrate and exhibits inhibitory activity against both LpxA and LpxD enzymes; however, it has not yet progressed to clinical evaluation (Jenkins et al., 2014). To our knowledge, there are no commercially available FDA-approved drugs targeting Lipid A biosynthesis.

#### 3.3.2 Core oligosaccharide biosynthesis

The core oligosaccharide is the bridging component of lipopolysaccharide (LPS) in *E.coli* and *Salmonella* species, situated between the hydrophobic Lipid A anchor and the O-antigen (**Figure 4C**). The formation of the LPS core oligosaccharide in Gram-negative bacteria (such as *E. coli* and *Salmonella*) is driven primarily by a dedicated cluster of glycosyltransferases, kinases, and modifying enzymes encoded by the *waa* family of glycosyl transferases and associated LPS biosynthesis proteins. WaaC and WaaF are sequential ADP-heptose-dependent glycosyltransferases involved in LPS inner-core assembly. WaaC adopts a GT-B fold comprising two Rossmann-like domains that form the donor- and acceptor-substrate binding cleft, with structural studies revealing conserved residues involved in substrate recognition and catalysis. WaaF is a structurally related heptosyltransferase that catalyzes the subsequent transfer of the second heptose residue, although its structural characterization is less extensive than that of WaaC. Together, their sequential catalytic activities establish the heptose backbone of the LPS inner core (Grizot et al., 2006) that provides the attachment site for the O-antigen polymer.

#### 3.3.3 O-antigen (somatic antigen) biosynthesis

Unlike the highly conserved lipid A and core oligosaccharide, the O-antigen exhibits extensive structural diversity owing to variations in its sugar composition, glycosidic linkages, and chain length. This diversity is generated by the coordinated action of membrane-associated biosynthetic proteins, including the flippase Wzx, the polymerase Wzy, the chain-length regulator Wzz, and the ligase WaaL (Islam and Lam, 2014), (**Figure 4C**) (De Castro et al., 2010). Wzx is a highly hydrophobic multi-pass inner-membrane flippase that translocates lipid-linked O-antigen units, whereas Wzy is an integral membrane polymerase that catalyzes repeat-unit polymerization on the periplasmic side (Islam et al., 2010). Wzz belongs to the polysaccharide co-polymerase family and forms oligomeric assemblies that regulate the modal O-antigen chain length. WaaL is a GT-C-family membrane glycosyltransferase containing 12 transmembrane helices and a conserved periplasmic catalytic region that ligates the completed O-antigen polymer to the lipid A core (Weckener et al., 2023). More than 180 O-serotypes have been identified in *E. coli* and 46 known O-antigens in *S. enterica*. Among them, O12 is considered the backbone of all 3 types of strains, whereas O2 is for *S.* Paratyphi, O9 in *S*. Typhi and O4 in *S*. Typhimurium. The O-antigen represents the most antigenically diverse region of the bacterial cell surface and forms the basis of serotyping schemes used to distinguish bacterial strains.

The resulting O-antigen architecture governs outer membrane stability, immune evasion, and resistance to complement-mediated killing, highlighting these biosynthetic proteins as promising targets for structure-guided antimicrobial intervention (Marolda et al., 2006). The O:2 polysaccharide-CRM197 conjugate preserved the native O:2 epitope while incorporating novel terminal KDO residues. It induced robust functional immune responses in rabbits, with sera demonstrating strong bactericidal activity against multiple *S*. Paratyphi A clinical isolates, indicating its efficacy as a broadly protective vaccine candidate. (Micoli et al., 2012). The repeating oligosaccharide units of O-antigen are readily recognized by the host immune system enabling serovar-specific identification. Thus, O-antigen represents an attractive vaccine and diagnostic target owing to its serovar-specific antigenic properties (Siba et al., 2012).

#### 3.3.4 Vi antigen (Typhi-specific)

**Figure 5:**
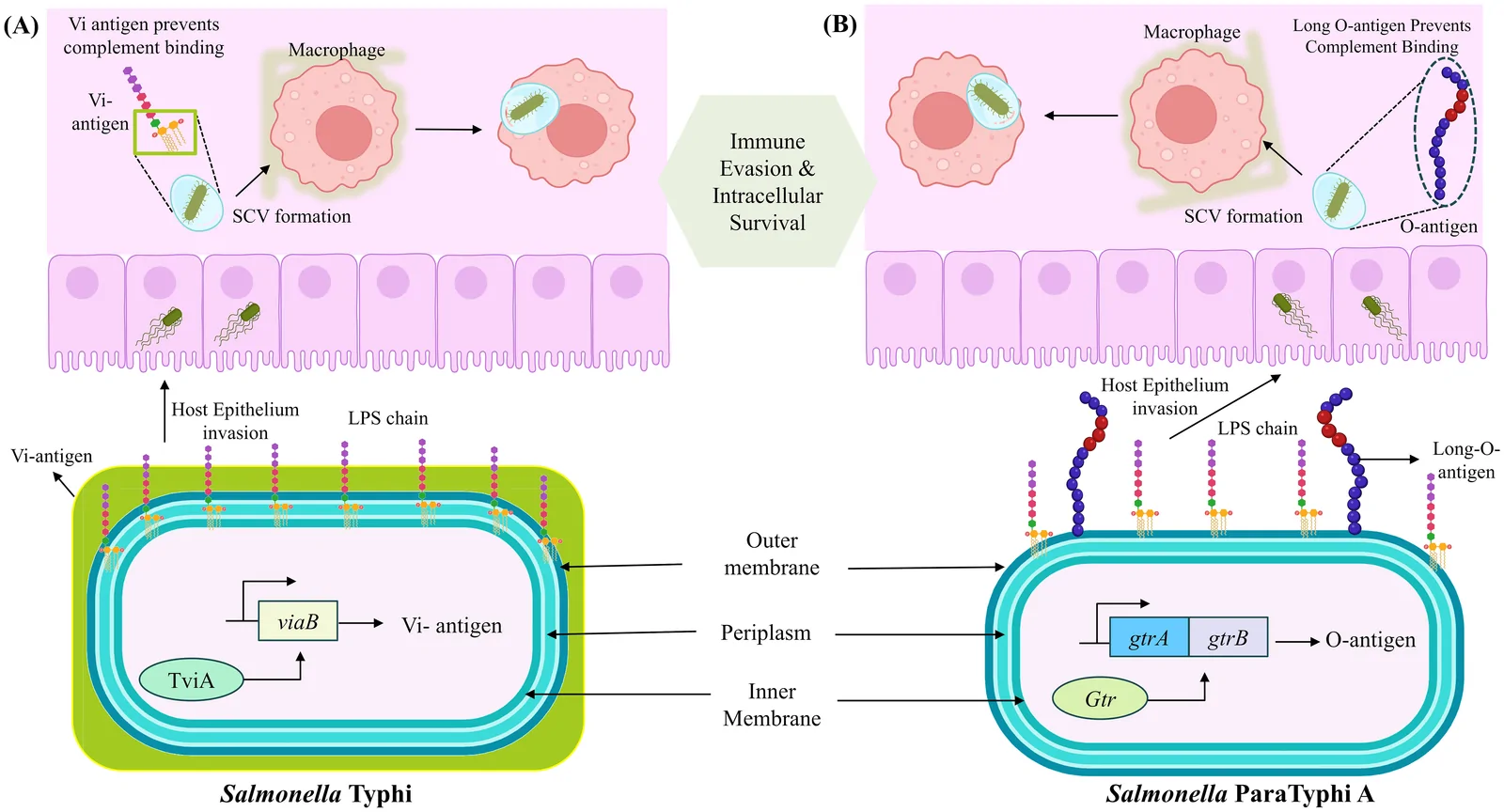
Structural differences and virulence-associated surface features of *Salmonella* Typhi and *Salmonella* Paratyphi A. **(A)** *Salmonella* Typhi possesses the Vi capsular polysaccharide (Vi antigen), which masks underlying surface antigens, reduces complement deposition and immune recognition, and promotes intracellular survival within host cells. **(B)** *Salmonella* Paratyphi A lacks the Vi capsule but exhibits a modified and extended O-antigen polysaccharide structure that contributes to immune evasion, resistance to host defence mechanisms, and persistence within intracellular environments. These distinct surface adaptations enable both serovars to establish systemic infection despite differences in their virulence strategies.

The Vi capsular polysaccharide (Vi antigen) is a key virulence structure that distinguishes *S*. Typhi from other serovars. It allows bacteria to combat complement-mediated killing and survive inside macrophages. It is synthesised by specific biosynthetic enzymes TviBCDE (polymer assembly enzymes). TviA protein is a DNA-binding response regulator that controls the switch between invasive and persistent phenotypes. It initiates the production of all subsequent biosynthetic and transport enzymes when environmental signals demand capsule formation (Winter et al., 2009). TviB functions as a UDP-N-acetylglucosamine (UDP-GlcNAc) 6-dehydrogenase. It catalyzes the NAD-dependent oxidation of UDP-GlcNAc at the C-6 position to generate the intermediate nucleotide sugar UDP-N-acetylglucosaminuronic acid (UDP-GlcNAcA). This chemical conversion provides the fundamental carboxylated sugar foundation required to eventually build the unique Vi antigen polymer. TviC works immediately downstream of TviB as a UDP-GlcNAcA 4-epimerase. It inverts the hydroxyl configuration at the C-4 position of the intermediate to produce UDP-N-acetylgalactosaminuronic acid (UDP-GalNAcA), the essential monomer unit for polymer assembly. By altering this specific stereochemistry, it successfully generates the active nucleotide-sugar precursors needed for the polymerization reaction (Zhang et al., 2006). TviE is a glycosyltransferase that utilizes the UDP-GalNAcA precursors synthesized by TviB and TviC. It catalyzes the progressive formation of α-1,4-glycosidic bonds to assemble the linear homopolymer chain of the Vi antigen. TviD is an O-acetyltransferase that structurally modifies the growing carbohydrate chain. It transfers acetyl groups from acetyl-CoA to the C-3 position of the GalNAcA residues, a structural alteration that is critical for immunogenicity and resistance to serum killing (Wear et al., 2022). The Vi antigen is exported by vexABCDE (ABC transporter and anchoring proteins), which are regulated by the viaB locus on SPI-7 from which vexA-D prevents the lethal intracellular accumulation of the large polymer by actively pumping the completed chain out to the bacterial cell surface (Wetter et al., 2012). VexE functions as an acyltransferase located at the outer membrane. It attaches a phospholipid or fatty acid anchor to the terminal end of the exported Vi polysaccharide, securing the protective capsule to the external surface of the bacterium. This final modification prevents the Vi antigen from shedding into the surrounding medium, ensuring it remains a functional surface shield against host immune responses (Wilson et al., 2011). Although the Vi antigen is absent in *S.* Paratyphi A, it is replaced by an elongated O-chain to confer immune resistance (**Figure 5B**; **Table 4**) (T. Saha et al., 2023). The Vi capsule is an attractive vaccine and therapeutic target because inhibition of the *viaB*-encoded biosynthetic pathway or *viaA*-mediated regulation abolishes capsule production, thereby enhancing bacterial susceptibility to host immune clearance, while the surface-exposed Vi polysaccharide is the basis of licensed typhoid conjugate vaccines and serological diagnostics (Snellings et al., 1977). The surface-exposed Vi polysaccharide forms the antigenic basis of licensed typhoid conjugate vaccines, including Vi–CRM197 formulations, and is also used as a target for serological detection and immune-response monitoring (Micoli et al., 2011). From recent studies, to facilitate multivalent immunization, a genetically altered *S.* Paratyphi A strain co-expressing Vi and O:2 antigens was designed by incorporating the viaB locus from *S.* Typhi. The GMMA particles (72–83 nm) were constructed to carry both antigens without structural hindrance. Vaccination induced strong antibody responses capable of killing *S.* Paratyphi A and Vi-expressing Citrobacter freundii, making GMMA an attractive dual-action platform for both typhoid and paratyphoid fever (Gasperini et al., 2021).

**Table 4:** Comparative protein features of *S.* Typhi and *S.* Paratyphi A.

Feature	*S.* Typhi	*S.* Paratyphi A	Functional implication
Capsule	Vi capsule present (viaB locus)	Capsule absent	Enhanced immune evasion in *S.* Typhi (Kaur and Jain, 2012),
Vaccines	Vi vaccines	O2 + CRM197 conjugate vaccines	Differential antigenicity and diagnostics (Micoli et al., 2012; Pinto et al., 2025)
T3SS regulation	Moderate SPI-1 activation	Reduced SPI-1 activation	Altered invasion efficiency (Johnson et al., 2017)
Motility	Limited intracellular motility	Retains intracellular motility	Differences in intracellular dynamics (Fass and Groisman, 2009).
Adhesins	FimH, T2544 dominant	T2544, StkF prominent	Variation in host interaction (Wagner and Hensel, 2011; Kolenda et al., 2019; Uchiya et al., 2019)
Toxin system	Classical CdtB-PltA-PltB	Variant PltC present	Possible differences in toxin delivery (Chang et al., 2022; Song et al., 2013; Fowler et al., 2019)
Immune evasion	Vi Capsule-mediated	O-antigen-mediated	Distinct complement resistance mechanisms (Wang et al., 2020).

#### 3.3.5 O-Antigen modification in *S*. Paratyphi A

The absence of the Vi capsule forces *Salmonella* Paratyphi A to rely on specific, modified O-antigen structures for survival (**Figure 5B**). O-antigen modification involves glucosylation mediated by GtrA and acetylation mediated by GtrB, while the O-antigen backbone contains mannose, rhamnose, and galactose residues. These protein-mediated modifications alter O-antigen structure and may affect serum resistance, immunological evasion, and antibody identification, thereby diminishing vaccination effectiveness (Kintz et al., 2017) (**Figure 5B**; **Table 4**). The O-antigen in *S*. Paratyphi A is considered an attractive vaccine target because it is the outermost and highly exposed component of lipopolysaccharide with specific modifications, making it readily specific towards *S*. Paratyphi A (Mastroeni and Rossi, 2020).

### 3.4 Intracellular stress-related signalling molecules

#### 3.4.1 Master regulators

**Figure 6:**
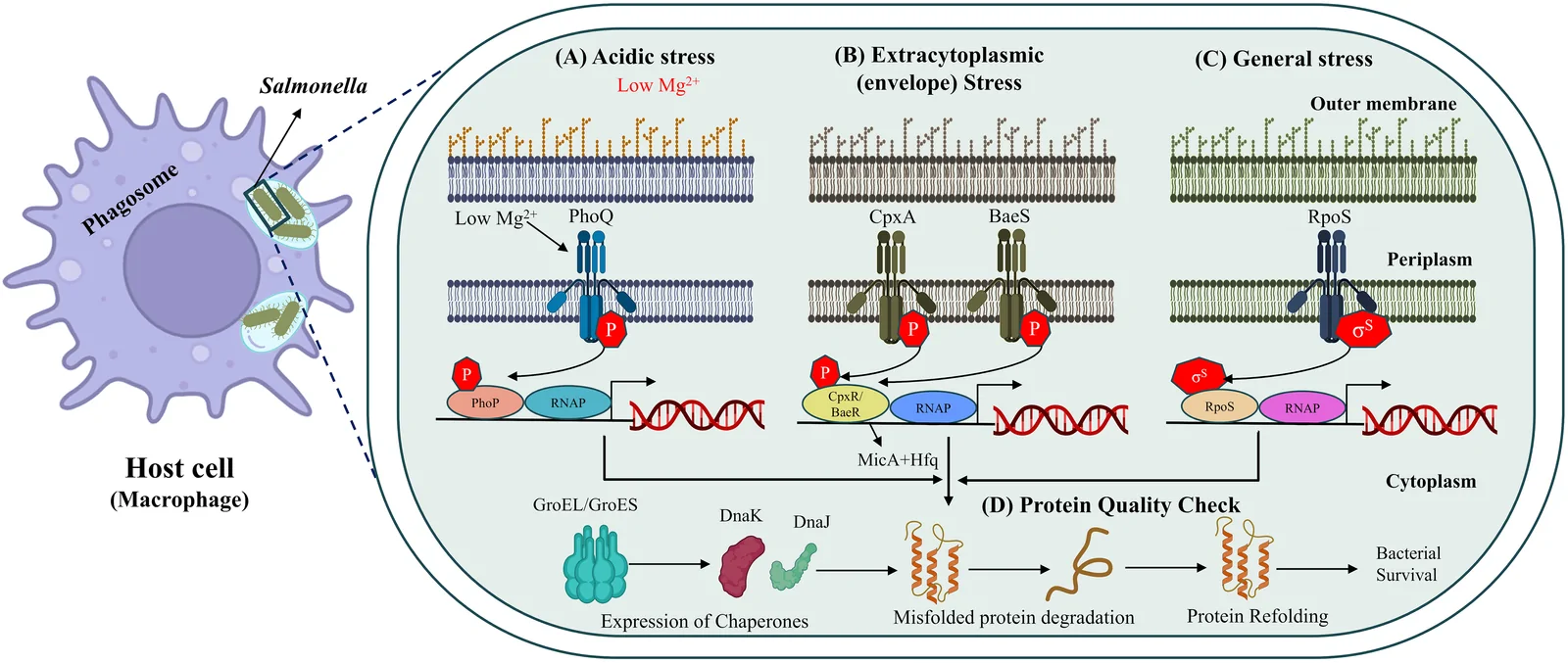
Stress-response mechanisms promoting intracellular survival of *Salmonella* within macrophages. **(A)** Acidic and low-Mg²^+^ stress: The PhoP/PhoQ two-component system senses acidic conditions and Mg²^+^ limitation and activates PhoP-dependent transcription of stress-adaptation genes. **(B)** Envelope stress: The CpxA/CpxR and BaeS/BaeR systems detect envelope perturbations and regulate genes involved in envelope homeostasis and stress adaptation, with MicA-Hfq contributing to post-transcriptional regulation. **(C)** General stress response: RpoS (σ^S) redirects transcription toward stress-responsive genes, enabling adaptation to the intracellular environment. **(D)** Protein quality control: Stress-induced chaperones, including GroEL/GroES, DnaK, and DnaJ, promote protein folding and recovery from protein damage, thereby supporting bacterial survival and persistence within macrophages.

Within the host cell environment, the bacteria have an elegant survival mechanism that involves oxidative stress sensors and regulators to evade host-related stress and survive within. For example, the bacterial two-component system includes the master response regulator PhoP and BaeSR (Sj et al., 2013), and the master sensor regulator PhoQ (E. Choi et al., 2009) that detects acidic/low-Mg²^+^ environments and regulates more than 40 genes related to antimicrobial peptide resistance, Vi expression, and survival in the host (Arricau et al., 1998). The PhoP/PhoQ two-component system provides a structurally characterized example of environmental sensing (**Figure 6A**). PhoQ is a homodimeric membrane-embedded histidine kinase comprising a periplasmic sensor domain, two transmembrane helices, an HAMP signal-transduction domain, a dimerization/histidine-phosphotransfer (DHp) domain, and a C-terminal catalytic ATP-binding domain (Mao et al., 2025). Its periplasmic sensor domain adopts a PDC-type fold with an acidic surface involved in divalent-cation sensing, whereas signal-induced conformational changes are transmitted through the transmembrane and HAMP regions to regulate kinase activity. PhoP contains an N-terminal receiver domain and a C-terminal DNA-binding domain; phosphorylation of the conserved receiver-domain aspartate promotes the active conformation required for binding PhoP-responsive promoters (Ishii and Eguchi, 2021). The other prevalent signalling mechanism involves CpxAR-MicA-Hfq, (**Figure 6B**) where the CpxAR system represents another envelope-stress signalling pathway. CpxA senses extracytoplasmic stress and transmits the signal through phosphorylation of CpxR, which subsequently modulates expression of stress- and virulence-associated genes. CpxA contains an extracytoplasmic sensor domain and a cytoplasmic HAMP–DHp–catalytic core, whereas CpxR comprises a receiver domain linked to a DNA-binding domain (Kurabayashi et al., 2014). In addition, the RNA chaperone Hfq forms a hexameric Sm-like scaffold that facilitates post-transcriptional regulation by small RNAs such as MicA. HlyE, in contrast, is a structurally distinct α-helical pore-forming toxin that can oligomerize into membrane pores, providing the molecular basis for its cytolytic activity (Moll et al., 2003). These master regulators play a critical role by initiating the downstream pathway in *Salmonella* towards pathogenesis, therefore targeted drug therapy against these master regulators might be more effective in controlling the pathogenesis.

PhoPQ and BaeSR are the attractive therapeutic targets because inhibition of these two-component systems disrupts virulence regulation, antimicrobial peptide resistance, and stress adaptation. Moreover, phoP/phoQ-deleted *Salmonella* strains are highly attenuated yet immunogenic and have been extensively developed as live oral vaccine candidates and antigen delivery vectors (Mao et al., 2025).

#### 3.4.2 Stress-response proteins

During the initial bacterial contact with the host, the major stress regulator genes and proteins, such as RpoS, PhoPQ, Fur, and OmpR/EnvZ, are expressed for survival against the host-related stress conditions (Rychlik and Barrow, 2005). For example, RpoS acts as the master regulator for the general stress response, preparing bacterial cells to survive nutrient deprivation, osmotic shock, acid, and oxidative stress within the host (**Figure 6C**) (Nickerson and Curtiss, 1997; Sheikh et al., 2010). Structurally, RpoS (σ^S^) is an alternative σ^70^ family sigma factor composed of conserved σ_2_ and σ_4_ regions connected by flexible linkers. The σ_2_ and σ_4_ domains interact with the core RNA polymerase and recognize promoter elements, while the σ_2_ region also provides an interaction surface for regulatory factors such as Crl. These domain-level interactions enable RpoS to redirect RNA polymerase toward stress-responsive promoters and thereby coordinate adaptation to acid, oxidative, osmotic and nutrient-limiting conditions (Cavaliere et al., 2018). Recently, epigallocatechin gallate (a potent antioxidant), Batimastat (a matrix metalloproteinase inhibitor (MMPI)) and BRITE-338733 (an inhibitor of bacterial RecA ATPase activity) tested as an RpoS, RpoE and RecA inhibitor, respectively. While epigallocatechin gallate and Batimastat showed no significant effects under the tested conditions, BRITE-338733 successfully disrupted the SOS response, reducing antibiotic tolerance in *Salmonella* (Hassanin et al., 2025). Researchers target specific domains of the PhoPQ complex using diverse chemical classes to disrupt bacterial virulence and restore antibiotic sensitivity. Hydrazones (such as compound A16B1) and quinazoline-based compounds represent two highly potent, non-cytotoxic classes of synthetic small-molecule inhibitors that combat bacterial virulence by blocking the PhoP/PhoQ two-component regulatory system (Cabezudo et al., 2022). Fur-related proteins play a critical role in iron-acquisition and survival within the host to maintain iron homeostasis, will be discussed in upcoming sections.

Furthermore, heat shock proteins such as, GroEL, DnaK function as essential molecular chaperones during host-associated stress. These proteins are upregulated under conditions such as high temperature, nutrient limitation, acidic pH within the host, in order to properly fold the critical bacterial proteins responsible for their survival (Tang et al., 1997) (**Figure 6D**). These protein chaperones enable proper protein folding and expression within the bacterium during the stress condition. Indeed, periplasmic serine protease/chaperone, htrA, when knock down in *S.* Paratyphi A weakened the bacterium’s stress tolerance and virulence, making it an ideal therapeutic target for targeted therapy against typhoid (Zhu et al., 2015).

### 3.5 Metabolic and iron-acquisition proteins

**Figure 7:**
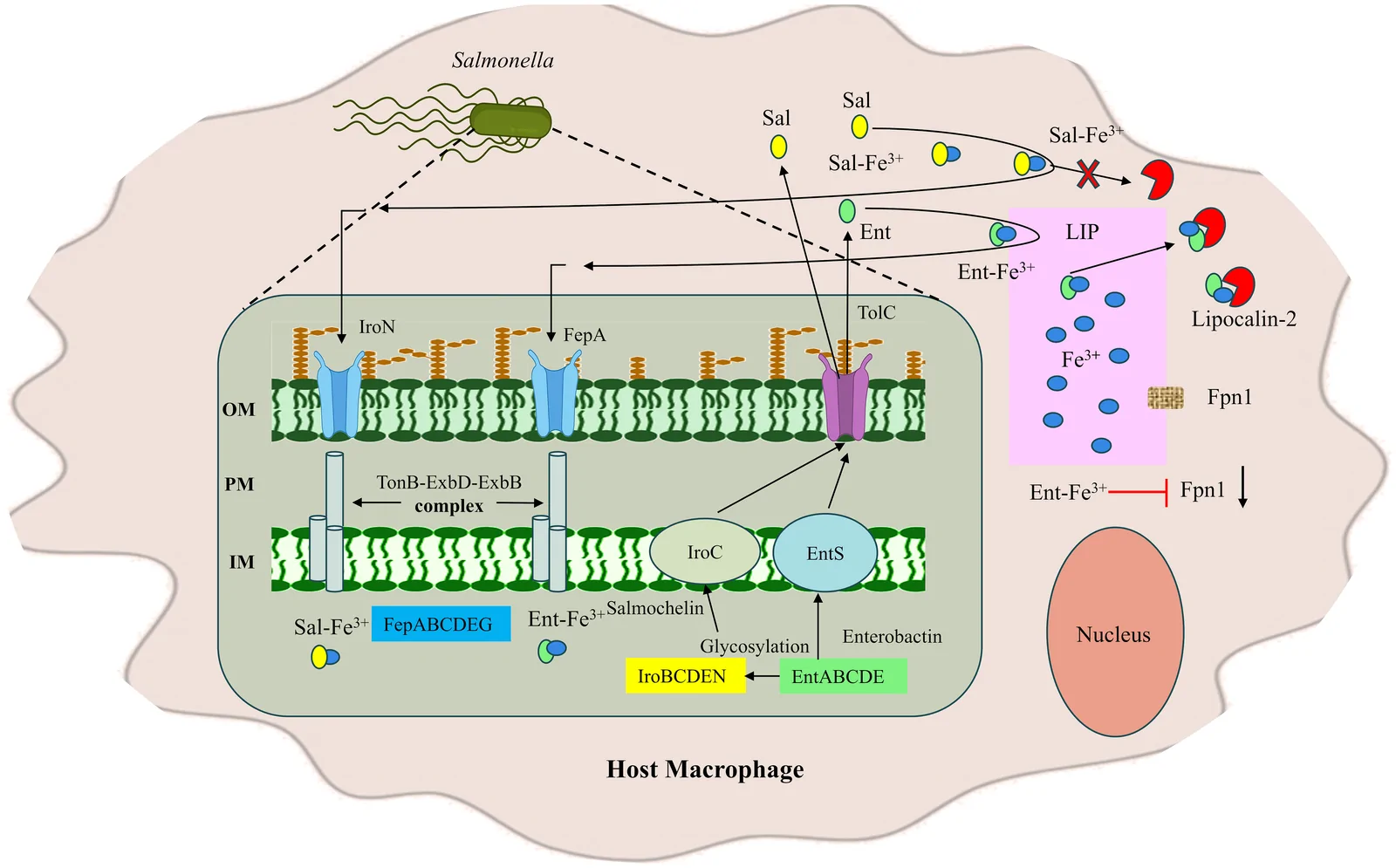
*Salmonella* iron acquisition pathway within host macrophages: During iron limitation inside host macrophages, *Salmonella* synthesizes the catecholate siderophore enterobactin through the EntABCDE biosynthetic pathway. Enterobactin scavenges ferric iron (Fe³^+^) from the host environment and facilitates its uptake into the bacterial cell through ferric-enterobactin transport systems. As a host defense mechanism, the siderophore-binding protein lipocalin-2 sequesters enterobactin and restricts bacterial iron acquisition. To overcome this nutritional immunity, *Salmonella* expresses the IroBCDEN gene cluster, which converts enterobactin into its glycosylated derivative, salmochelin. Salmochelin evades recognition by lipocalin-2, enabling continued iron acquisition and promoting bacterial survival and replication within macrophages.

For intracellular survival within the host, *Salmonella* needs iron for its nutrition and survival. Not surprisingly, iron uptake mechanisms play a critical role in the pathogenesis of infections in the *Enterobacteriaceae* family. Iron uptake systems are often distinguished by the production of a siderophore and the presence of OMPs that aid in its transport. In general, microbes synthesize and secrete low-molecular-weight siderophores to scavenge iron from the surrounding environment. The iron-bound siderophore then enters the bacteria through the designated OMP receptor with absolute specificity. *Salmonella* obtains ferrous iron via secreting the catecholate siderophores, such as Enterobactin (Ents) and Salmochelin (**Figure 7**) (Saha et al., 2018).

#### 3.5.1 Uptake of iron for intracellular survival

Enterobactin-mediated iron uptake is essential for *Salmonella* replication in macrophages and for colonization of systemic tissues in animal models. Enterobactin biosynthesis involves EntC, EntB and EntA in the synthesis of 2,3-dihydroxybenzoic acid, followed by EntB, EntD and EntE-mediated assembly of the enterobactin precursor. The biosynthesis of enterobactin in *Salmonella* occurs through a coordinated, multi-step cytoplasmic pathway comprising two major stages: the synthesis of 2,3-dihydroxybenzoic acid (DHB) and the subsequent non-ribosomal peptide synthetase (NRPS)-mediated assembly of enterobactin (Blahut et al., 2020). The first stage involves EntC, EntB, and EntA, which collectively synthesize DHB, while the second stage involves EntB, EntD, and EntE, which participate in the NRPS-dependent assembly of the enterobactin precursor (Amiri et al., 2025). The Ents chelates LIP (Labile iron pool) and forms an Ent-Fe complex (**Figure 7**). This complex downregulates ferroportin (Fpn1), which is considered the “exit door” for iron, preventing iron from leaving the cell. Subsequently, *Salmonella* produces a family of ferric-enterobactin uptake proteins (*FepABCDEG*) that mediate the transport of ferric-enterobactin into the cell. FepA, an outer membrane receptor, recognizes and binds the ferric-enterobactin complex extracellularly and facilitates its transport across the outer membrane. The complex is then captured by the periplasmic binding protein FepB and delivered to the inner membrane (Rabsch et al., 1999). FepC, FepD, and FepG form an ATP-binding cassette (ABC) transporter complex in the inner membrane, which uses ATP-derived energy to translocate ferric-enterobactin into the cytoplasm (Fernandez-Beros et al., 1989; Nagy et al., 2013; Jia et al., 2022). The host defence protein lipocalin-2 restricts bacterial iron acquisition by binding and accumulating Ents. To evade this mechanism, *Salmonella* expresses the *IroBCDEN* gene cluster for the synthesis of Salmochelin, a glycosylated derivative of enterobactin, which involves transfer of glucose residues from UDP-glucose to the C5 position of the 2,3-dihydroxybenzoylserine moieties of enterobactin by IroB (Motz et al., 2025). This structural alteration physically blocks the host immune protein lipocalin-2 from binding the siderophore. IroC acts as an ATP-binding cassette (ABC) transporter dedicated to pumping the newly synthesized apo-salmochelin trimers and dimers out of the cytoplasm into the periplasm, allowing them to ultimately exit into the extracellular space to scavenge ferric iron (Fe^3+^) (Caza et al., 2008). Once the iron-bound (ferric) salmochelin is brought back inside the cytoplasm, IroD hydrolyzes the ester bonds of the macrocyclic salmochelin backbone. This linearization and degradation structurally collapse the coordination complex, allowing the cell to reduce and utilize the trapped iron. IroE acts as a specialized “pre-processing” esterase. It performs a single-cut cleavage of the cyclic salmochelin trimer (S4) into its linear dimer or monomer forms (such as S2) (Lin et al., 2005). This structural linearization aids and optimizes subsequent transport steps across the inner membrane (Lin et al., 2005). Salmochelin collects iron from LIP and forms sal-fe complex which is recognized by IroN and transported back to the bacteria. Finally, the cellular absorption carried out by the Fep system (FepB and FepDGC) inside bacteria. It is critical for *Salmonella*’s ability to overcome host iron limitation and develop systemic infection (Foster and Hall, 1992).

#### 3.5.2 Proteins related to iron homeostasis

Excess iron accumulation in the cytoplasm is toxic to the bacterial cells; therefore, a stringent iron-regulation mechanism is critical for their survival. The Ferric Uptake Regulator (Fur) is the master transcriptional repressor that controls iron homeostasis and uptake in *Salmonella* (Troxell and Hassan, 2013). Fur (ferric uptake regulator) is a dimeric metalloprotein that controls iron homeostasis. Structurally, each monomer has an N-terminal DNA-binding winged helix domain and a C-terminal α/β dimerization domain, stabilized by a structural Zn(II) site (PDB: 1MZB) (Berg et al., 2020). Catalytically, Fur senses iron through a regulatory Fe(II) site (His86, Asp88, Glu107, His124), which modulates activity depending on iron availability. Its active site architecture positions DNA-binding helices into the major groove of target promoters, recognizing ~27–30 bp “Fur boxes.” This arrangement allows cooperative binding and DNA bending, enabling repression of iron-regulated genes (Pohl et al., 2003). Under iron-rich conditions, Fe²^+^-bound Fur binds Fur boxes and most commonly represses transcription by sterically occluding RNA polymerase, thereby suppressing the expression of high-affinity iron acquisition genes, including the *FepABCDE* ferric enterobactin transport system. Conversely, when intracellular iron becomes limiting, Fe²^+^ dissociates from Fur, reducing its DNA-binding affinity and leading to the release of Fur from operator DNA. (J. Choi and Ryu, 2019; Lim et al., 2020; Mey et al., 2021). This derepression permits the transcription of iron uptake and iron-scavenging systems, enabling *Salmonella* to restore intracellular iron homeostasis during host-mediated iron restriction (Foster and Hall, 1992).

Several studies have explored the Ferric Uptake Regulator (Fur) as a promising antibacterial target. Among these approaches, peptide aptamers have been developed to inhibit *Escherichia coli* Fur, with candidate inhibitors identified by screening a 20-million-member peptide library using a yeast two-hybrid assay (Abed et al., 2007). Among the three metal-binding sites of Fur, S2 and S3 are highly conserved across bacteria, whereas S1 is absent in some species lacking the requisite cysteine residues, reflecting evolutionary adaptations in DNA-binding regulation. Notably, the conserved S2 site represents an attractive therapeutic target, as small molecules or metal chelators can disrupt Fe²^+^ coordination, preventing the conformational reorientation of the DNA-binding domain (DBD) relative to the dimerization domain (DD) required for formation of the transcriptionally active Fe-Fur complex (Kang et al., 2024).

In the presence of oxygen or metabolic stress, loose, unshielded iron (Fe^2+)^ reacts with cellular byproducts to generate destructive free radicals via the Fenton reaction (Serebnitskiy et al., 2025), disrupting the bacterial cells. *S*. Typhimurium utilizes four distinct iron storage proteins to manage intracellular iron homeostasis, avoid toxicity, and protect against oxidative stress: Bacterioferritin (Bfr), Ferritin A (FtnA), Ferritin B (FtnB) and DNA-binding protein from starved cells (Dps) (Cunrath and Palmer, 2021). Bacterioferritin (Bfr) is a heme-containing protein that serves as the main iron-storage molecule in *Salmonella*. It is highly expressed under iron-rich conditions, where it sequesters excess iron and maintains iron homeostasis. Loss of bfr increases intracellular free iron levels and makes the bacterium more susceptible to hydrogen peroxide (H_2_O_2_)-induced oxidative stress (Yao et al., 2022) Ferritin A (FtnA) functions with Bfr as a major iron-storage protein under iron-rich conditions and is repressed by the Fur regulator during iron limitation. In contrast, Ferritin B (FtnB) is induced under low-iron conditions and serves as a readily available Fe²^+^ reservoir, supplying iron for the repair of oxidatively damaged iron-sulfur (Fe-S) clusters in metabolic enzyme (Bereswill et al., 2000).

A recently proposed therapeutic strategy targets the bacterioferritin (Bfr) and its physiological partner, bacterioferritin-associated ferredoxin (Bfd). Bfr maintains cytosolic iron homeostasis by oxidizing Fe²^+^ to Fe³^+^ and storing it within its internal cavity, while Bfd facilitates Fe³^+^ reduction and Fe²^+^ release. Disruption of the Bfr–Bfd interaction causes Fe³^+^ accumulation in Bfr and cytosolic iron depletion, impairing biofilm maintenance and promoting biofilm cell death (Rivera, 2023). Small-molecule inhibitors (such as derivatives of *4-aminoisoindoline-1,3-dione*)) targeting the Bfd-binding site of Bfr can therefore block iron mobilization and induce biofilm-associated cell death, highlighting the Bfr–Bfd complex as a potential therapeutic target (Soldano et al., 2021). Dps (DNA-binding Protein from Starved Cells) is a dodecamer ferritin-like protein that oxidizes Fe²^+^ at di-iron catalytic centers while its positively charged DNA-binding surface condenses and shields chromosomal DNA from hydroxyl radical-mediated damage (Orban and Finkel, 2022). It also sequesters free iron, preventing the formation of harmful hydroxyl radicals through the Fenton reaction. Expression of Dps is strongly induced under iron-limiting and oxidative stress conditions, contributing to bacterial survival (Karas et al., 2015; Khare et al., 2017). Blocking this protective mechanism renders *Salmonella* hypersensitive to host-immune attacks, destroying its tolerance to hydrogen peroxide (H_2_O_2_) and causing lethal DNA fragmentation (Halsey et al., 2004) This structural and functional breakdown drastically decreases the pathogen’s survival rate inside host macrophages, providing a highly effective therapeutic strategy to clear stubborn infections (Karas et al., 2015). In summary, *Salmonella* employs a highly sophisticated strategy for maintaining iron homeostasis. To maintain iron homeostasis, the Fur regulon plays a critical role in controlling the entire network of iron uptake, transport, storage, and intracellular metabolism. As a part of the host defence mechanism, *Salmonella* successfully evades the iron-sequestering activity of lipocalin-2 by developing alternative pathways for iron acquisition to support its nutritional demands. Iron-acquisition and homeostasis can be hindered in *Salmonella* by targeting any of the above suitable drug targets to induce iron-deprived infection control or promote bacterial toxicity through disruption of iron homeostasis.

#### 3.5.3 Iron-sulfur cluster biogenesis

**Figure 8:**
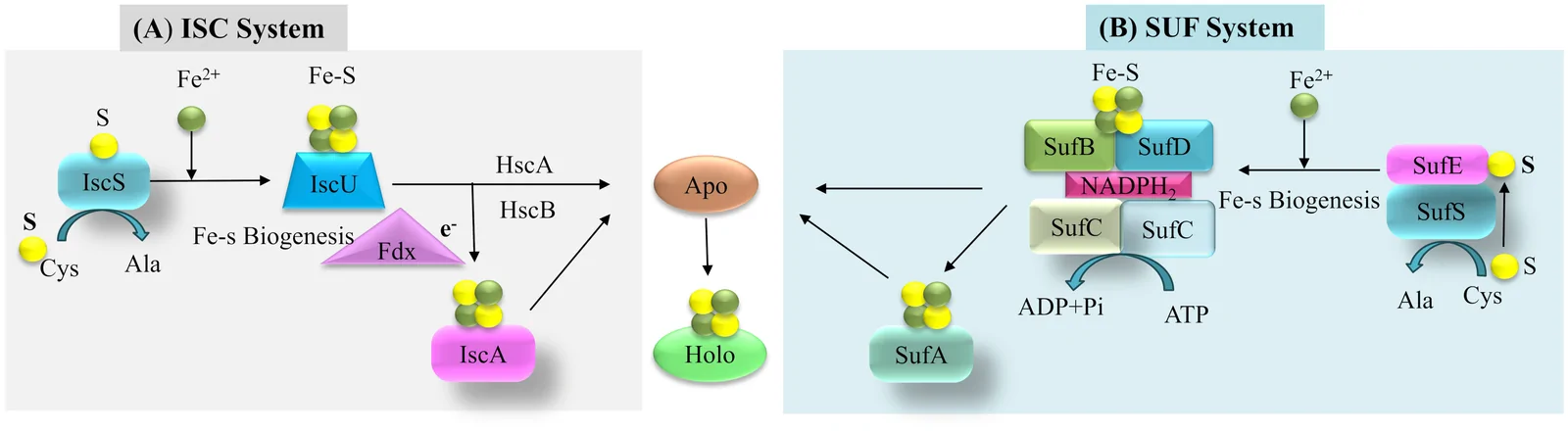
Comparison of the ISC and SUF pathways for iron–sulfur (Fe–S) cluster biogenesis in *Salmonella*. **(A)** ISC (Iron–Sulfur Cluster) system: Under normal growth conditions, the cysteine desulfurase IscS mobilizes sulfur from cysteine, which is assembled with ferrous iron (Fe²^+^) on the scaffold protein IscU to form Fe–S clusters. Cluster assembly is supported by ferredoxin (Fdx), while the chaperones HscA and HscB facilitate cluster maturation and transfer to recipient proteins directly or via IscA, generating functional holo-proteins. **(B)** SUF (Sulfur Utilization Factor) system: Under iron limitation and oxidative stress, the SUF pathway serves as a stress-resistant Fe–S cluster assembly system. Sulfur mobilized by the SufS–SufE complex and iron are assembled on the SufBC_2_D scaffold complex in an ATP-dependent manner. The carrier protein SufA subsequently transfers Fe–S clusters to target apo-proteins, ensuring protein maturation and bacterial survival under hostile intracellular conditions.

Further, to maintain iron homeostasis in *Salmonella*, the ISC (Iron-Sulfur Cluster) and SUF (Sulfur Utilization Factor) systems form a complementary, dual-pathway network designed to ensure uninterrupted Fe-S cluster biogenesis across fluctuating environmental conditions (Roberts et al., 2017) (**Figure 8**). The ISC (Iron-Sulfur Cluster) system is the primary pathway used by *Salmonella* and many other bacteria to assemble iron-sulfur (Fe-S) clusters, which are essential cofactors for proteins involved in respiration, DNA repair, metabolism, and regulation (Myriam et al., 2022). Under normal conditions, the pathway initiates with IscS, a cysteine desulfurase that transfers sulfur to the IscU scaffold protein, where transient Fe-S clusters are built and assembled with electrons provided by Ferredoxin (Fdx) (Urbina et al., 2001). Utilizing energy from ATP hydrolysis, co-chaperones HscA (Hsp70-class) and HscB (Hsp40-class) bind to IscU and induce a conformational shift that forces the release of the fragile cluster. The mature Fe-S cluster was then being escorted by carrier protein IscA directly to target apo-enzymes required for cellular respiration, DNA repair, and central metabolism (**Figure 8A**) (Ding, 2025). Researchers have found a way to inhibit IscS (cysteine desulfurase) or IscA, thereby completely blocking downstream cluster production, effectively starving the bacterium of its respiratory capacity. For instance, Copper (Cu I) toxicity actively disrupts bacterial growth by binding directly to the iron-binding site of IscA, blocking [4Fe-4S] cluster maturation and highlighting IscA as a druggable vulnerability (Tan et al., 2014).

However, because the crucial IscU scaffold relies on hypersensitive cysteine residues, the ISC system is rapidly inactivated by iron starvation and oxidative stress, thereby destroying nascent clusters and generating toxic free radicals. To mitigate this, bacteria induce the stress-resistant SUF (Sulfur Utilisation Factor) pathway (Suf ABCDSE) to preserve essential iron-sulfur cluster biogenesis (**Figure 8B**) (Esquilin-Lebron et al., 2021). The pathway begins with a multi-protein desulfurase complex (SufS and SufE). SufS extracts elemental sulfur from L-cysteine. The sulfur is then transferred to SufE, which functions as a specialized sulfur-acceptor mediator. Unlike the single-subunit IscU platform, the SUF system utilizes a larger, heterotrimeric or heterotetrameric complex to construct the nascent cluster, which begins with SufB, serves as the primary *de novo* scaffold containing specific, protected cysteine pockets that receive sulfur from SufE and binds scarce ferrous iron (Fe^2+^) (Blahut et al., 2020). SufD coordinates structurally with SufB to optimize iron binding. SufC is an active cytoplasmic ATPase working in pairs (SufC_2_) that couples ATP hydrolysis within the SufB framework and synthesizes new cluster configuration without needing standard chaperone assistance (Roberts et al., 2017; Blahut et al., 2020). Once synthesized, clusters are carried by carrier protein SufA, which behaves as an emergency molecular courier, bypassing the damaged ISC machinery to target and safely load the reactive cofactor directly into essential metabolic apo-enzymes (**Figure 8B**) (Outten, 2015). Researchers found novel small-molecule inhibitors through High-throughput screening libraries (like the GSK anti-tuberculosis library) are being used to discover small molecules (such as the compound ‘882) that specifically block the SufBCD complex to starve bacteria of energy (Choby et al., 2016).

To counter the vulnerability of the ISC system, *Salmonella* contains both ISC and SUF system for iron-sulfur cluster biogenesis. Especially, during iron starvation and oxidative stress, *Salmonella* induces the stress-resistant SUF pathway as an alternative Fe-S cluster biogenesis mechanism. The SUF machinery assembles and delivers Fe-S clusters using protected scaffold proteins and ATP-dependent assembly components, ensuring continued cofactor production and metabolic activity under hostile intracellular conditions.

### 3.6 *Salmonella* pathogenesis: typhoid toxin and export architecture

**Figure 9:**
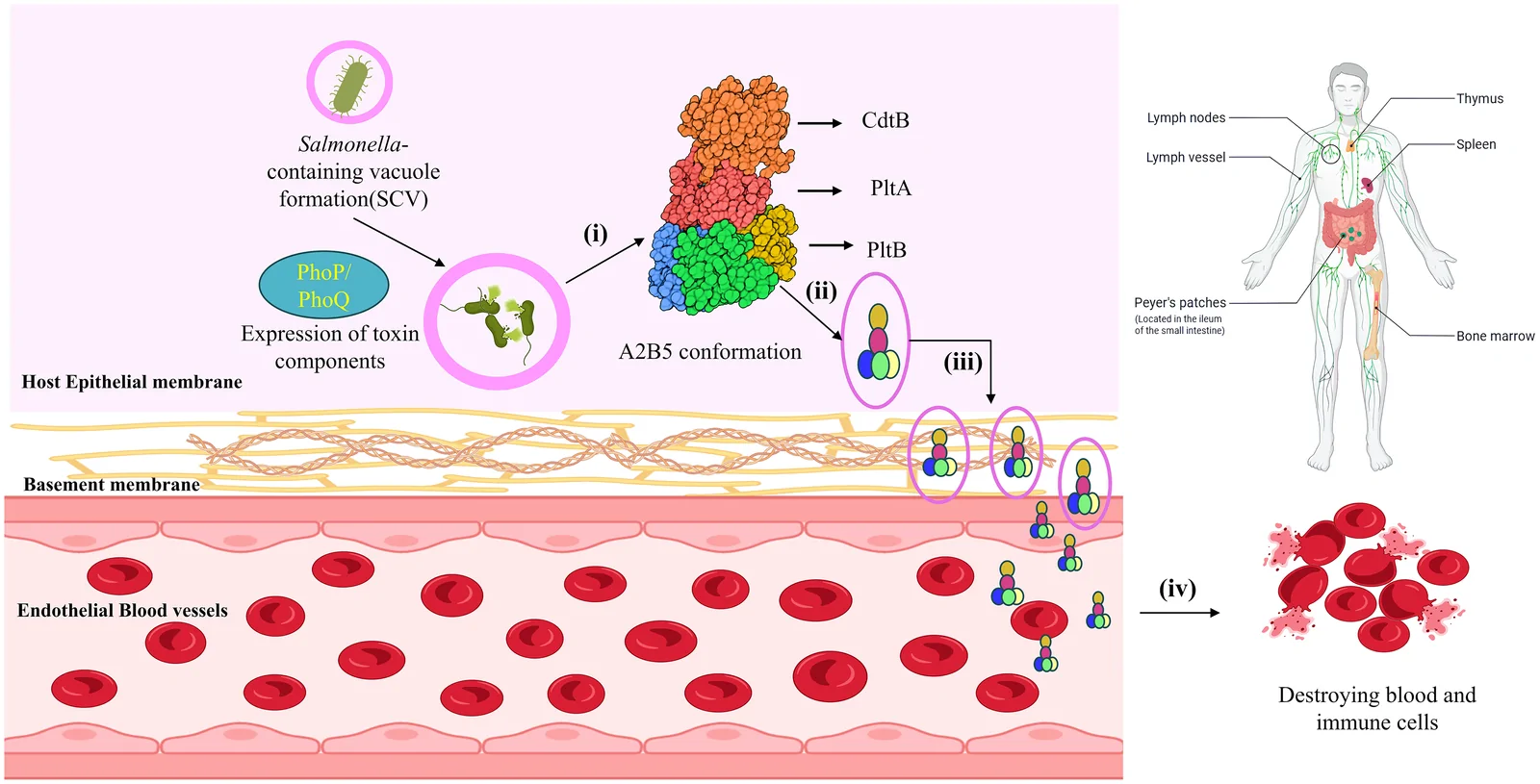
Production, assembly, and host-cell trafficking of the typhoid toxin (TT) during *Salmonella* Typhi infection. Within infected host cells, S. Typhi synthesizes the typhoid toxin subunits (CdtB, PltA, and PltB), which assemble into the active A_2_B_5_ toxin complex. The TT produced by *Salmonella* undergoes the following stages towards infection and pathogenesis in host cells. (i) they are produced intracellularly within the SCV; (ii) the PltB subunit of the toxin specifically recognizes acetyl neuraminic acid (Neu5Ac)-terminated sialoglycans, which are almost unique to the human host, and packs the toxins into vesicle carrier intermediates inside the SCV; (iii) the vesicles containing typhoid toxin enter the bloodstream and destroy blood cells and host immune cells; (iv) circulating typhoid toxin binds to susceptible host cells, including immune cells and blood cells, leading to cellular dysfunction, DNA damage, immune modulation, and contributing to the systemic manifestations of typhoid fever.

The typhoid toxin (TT) is produced only when *S.* Typhi enters host cells (**Figure 9**). Increased oxidative stress conditions such as low pH (acidic environment), low concentrations of essential ions (such as Mg^2+^ and phosphate) within the host cell are sensed by the two-component regulatory PhoP/PhoQ system, which subsequently leads to the expression of toxin gene (Del Bel Belluz et al., 2016) (**Figure 9**). The toxin genes *cdtB*, *pltA*, and *pltB* are co-transcribed as an operon. The *cdtB-pltA-pltB* operon encodes the A_2_B_5_ typhoid toxin, a highly stable heteroheptamer comprising the catalytic A subunits CdtB (DNase) and PltA (ADP-ribosyltransferase), assembled onto a homopentameric PltB receptor-binding platform (PDB: 4K6L) (Chang et al., 2022). CdtB contains a divalent metal ion-dependent nuclease active site, whereas PltA catalyzes NAD^+^-dependent ADP-ribose transfer through a conserved catalytic pocket (Lara-Tejero and Galán, 2001). The CdtB subunit is the active, toxic component of the cytolethal distending toxin (CDT) produced by various pathogenic Gram-negative bacteria. It functions primarily as a metal-dependent nuclease (homologous to mammalian DNase I) and dual-activity phosphatase, inducing host DNA double-strand breaks and cell cycle arrest (Pons et al., 2019). Specifically, H160 acts as the core catalytic residue driving phosphodiester bond hydrolysis, while D273 coordinates the essential divalent metal ions required for enzyme activation. Concurrently, residues R117, R144, and N201 form a precise binding pocket that anchors host DNA, positioning it for target cleavage (PDB: 2F1N) (Pons et al., 2019). Within the *Salmonella*-containing vacuole (SCV), the subunits assemble through high-affinity protein–protein interfaces stabilized by an essential intermolecular disulfide bond between PltA and CdtB, while the pentameric PltB mediates multivalent glycan recognition, enhancing receptor avidity and efficient toxin trafficking to host cells (PDB: 6P4R) (Lee et al., 2020). These subunits are secreted into the lumen of the SCV, where they spontaneously assemble into the unique (A_2_B_5_) holotoxin structure (CdtB-PltA-PltB). The expression and transport of the TT represent a highly sophisticated survival and dissemination strategy used by typhoidal *Salmonella.* These TTs are secreted into the SCV and subsequently exported by vesicle carriers to the extracellular space (Johnson et al., 2018a) (**Figure 9**).

The TT produced by *Salmonella* undergoes the following stages towards infection and pathogenesis in host cells. Firstly, they are produced intracellularly within the SCV (**Figure 9** (i)); secondly, the PltB subunit of the toxin specifically recognizes acetyl neuraminic acid (Neu5Ac)-terminated sialoglycans, which are almost unique to the human host, and packs the toxins into vesicle carrier intermediates inside the SCV (**Figure 9** (ii)) (Chang et al., 2022). Thirdly, the toxin-containing vesicles are subsequently released into the extracellular environment and can reach distant susceptible host cells, where toxin internalization produces its characteristic cellular effects (**Figure 9** (iii and iv)) (Song et al., 2013; Fowler et al., 2019). The toxin’s packaging requires the COPII coat protein and the Sar1 GTPase along with the mannose-6-phosphate receptors (Kasberg et al., 2023) in *Salmonella.* Taken together, these protein systems function not as isolated determinants but as a coordinated and dynamic network that governs the progression of infection. The development of targeted human monoclonal antibodies (hmAbs), such as TyTx11 capable of binding to the catalytic sites of the CdtB subunit and TyTx4 that, binds with high affinity to PltB receptor-binding subunits, preventing the toxin from anchoring onto the host’s surface sialic acids. These mAbs act as highly specific anti-toxin therapies to neutralize severe manifestations, such as typhoid encephalopathy (Ahn et al., 2021).

CdtB is an excellent vaccine antigen and a therapeutic target for neutralizing antibodies. Preclinical studies have demonstrated that recombinant CdtB-based subunit vaccines can elicit robust neutralizing antibody responses. In addition, the development of humanized monoclonal antibodies that target CdtB provides a potential approach for adjunctive therapy and toxin neutralization (Raeisi et al., 2022). Protective antibodies targeting PltB can inhibit typhoid toxin function by disrupting its assembly and host-cell binding. The toxin requires the weak PltA-PltB interaction to form a functional holotoxin. Anti-PltB monoclonal antibodies can interfere with this interface, preventing holotoxin formation and blocking PltB recognition of sialoglycans on human cells, thereby inhibiting toxin entry and cellular damage (Ahn et al., 2021). Researchers designed camelid single-domain (VHHs) against typhoid toxin and identified 41 candidates targeting PltB or CdtB. Three antibodies, T2E7, T2G9, and T4E5, conferred substantial protection in mice, resulting in 100% survival following a lethal toxin dose (Dulal et al., 2021).

## 4 Comparative features of *Salmonella* Typhi and Paratyphi A

While *S.* Typhi has been extensively studied due to its association with classical typhoid fever, increasing evidence highlights the growing clinical relevance of *S.* Paratyphi A. Although both serovars share core virulence determinants, differences in capsular composition, effector repertoires, and regulatory mechanisms suggest distinct strategies of host adaptation (**Table 4**). As mentioned in section 3.3.5, notably, the absence of the Vi capsule in *S.* Paratyphi A is compensated by modifications in O-antigen structure and alternative immune evasion strategies (Kaur and Jain, 2012; Wetter et al., 2012) These differences have important implications for diagnostics, vaccine design, and therapeutic targeting, underscoring the need for a more balanced investigation of both serovars. Development of vaccines against *S.* Typhi can be based on Vi-based antigen, whereas *S.* Paratyphi A requires alternative strategies, such as O2 (O-antigen) conjugate vaccines, due to its different antigenic profile (Micoli et al., 2012; Pinto et al., 2025).

Functionally, both serovars show differences in invasion, motility, and host interaction. While several studies suggest differences in SPI-1 expression and invasion dynamics between S. Typhi and S. Paratyphi A, *S.* Paratyphi A shows reduced SPI-1 activity, suggesting differences in epithelial entry efficiency (Johnson et al., 2017). In terms of intracellular behaviour, *S.* Typhi has more limited motility, whereas *S.* Paratyphi A retains intracellular motility, which may influence how it spreads within host cells (Fass and Groisman, 2009; Stepien et al., 2024). Variations are also seen in adhesins, with *S.* Typhi primarily expressing FimH and T2544, while *S.* Paratyphi A expresses T2544 along with StkF, reflecting differences in host cell attachment mechanisms (Wagner and Hensel, 2011; Kolenda et al., 2019; Uchiya et al., 2019). Additionally, toxin systems differ slightly, while *S.* Typhi produces the classical CdtB-PltA-PltB typhoid toxin, *S.* Paratyphi A includes a variant component (PltC), which may alter toxin delivery and function (Chang et al., 2022; Song et al., 2013; Fowler et al., 2019). PltC shares 30–35% identity with PltB and exhibits different glycan-binding specificity. Recent studies from Jorge E. Galán et al., demonstrated that PltC can assemble with the A subunits to form an alternative toxin complex CdtB-PltA-PltC heteroheptamer, where the receptor-binding pentamer is composed of PltC rather than PltB in *S.* Paratyphi A (Galán, 2026). This distinctive characteristic may provide opportunities for the development of serovar-specific diagnostic and therapeutic strategies, although further experimental validation is required. These serovar-specific structural differences should be considered when designing diagnostic antibodies, aptamers, vaccine antigens, and anti-virulence agents, because targeting conserved proteins may provide broad coverage whereas targeting variable surface or receptor-binding proteins may enable serovar discrimination.

## 5 Protein-centric theragnostic opportunities

For therapeutic intervention against typhoidal *Salmonella*, several protein-centred strategies have emerged. Anti-virulence approaches focus on inhibiting the T3SS, with compounds targeting SipD and fluorothiazinon demonstrating the ability to suppress effector secretion and intracellular bacterial survival. Additional targets include metabolic and persistence-associated proteins such as WcaB, biofilm regulators (AdrA, BcsA, and CsgD), which offer opportunities for small-molecule inhibitors, vaccine development, and biomarker discovery. Toxin-neutralizing strategies are directed against the typhoid toxin components CdtB and PltB, where recombinant vaccines and monoclonal antibodies can block toxin assembly, receptor binding, and cellular entry (Chang et al., 2019; Jiao et al., 2020). Furthermore, disruption of critical toxin-host interactions, such as binding to sialoglycan mimic receptors, represents an additional therapeutic avenue (Marondedze and Govender, 2022).

Beyond treatment, toxin-specific antibodies and toxin detection assays also have potential diagnostic applications for identifying infection and exposure (Sesardic et al., 2004). Neutralizing monoclonal antibodies (mAbs) are beneficial for both therapeutics and diagnostics, making them useful for accurately identifying and collecting them in diagnostic testing. Overall, targeting secretion systems, metabolic pathways, iron-scavenging mechanisms, and toxin activity provides a multifaceted framework for the development of next-generation therapeutics against typhoidal *Salmonella*.

Protein-based diagnostics are emerging as powerful tools for the detection of enteric fever by enabling direct identification of pathogen-specific antigens in accessible clinical samples such as blood and faeces. Among these, Hemolysin E (HlyE) is an immunogenic protein expressed by *S.* Typhi and *S.* Paratyphi A and has emerged as a promising biomarker for enteric fever, while the LPS O-antigen remains a widely used immunogenic target despite limitations arising from cross-reactivity. Recent advances in aptamer-based technologies, including AptHlyE97, S-PS8.4, and Apt22, have demonstrated high affinity, specificity, thermal stability, and cost-effectiveness for detecting typhoidal *Salmonella* and differentiating it from non-typhoidal strains (Ahmad Najib et al., 2024; Pan et al., 2005; Rm et al., 2020). Antibody-based platforms such as ELISA and lateral-flow assays continue to target HlyE, LPS, vi polysaccharide, and outer membrane proteins, forming the basis of several rapid diagnostic systems. Integration of these protein targets into biosensor platforms has further improved the potential for rapid, field-deployable, and real-time diagnosis, particularly in resource-limited settings (Olsen et al., 2004).

In spite of substantial advances in technology, fundamental challenges remain. Culture-based strategies, while considered the diagnostic gold standard, have moderate sensitivity and a high rate of false negatives. Serological tests are limited by cross-reactivity due to common antigens. The Widal test has low sensitivity and specificity, whereas other rapid diagnostic tests can offer faster results but are restricted in their capacity to distinguish acute from prior diseases (**Table 5**).

**Table 5:** Protein-based diagnostic targets and platforms.

Target proteins	Detection strategy	Platform type	Preclinical/clinical trials	Diagnostic advantage
FimH	FimH–mannose recognition	Mannose-functionalized nanozyme biosensor (Man-MPN)	*In vitro* analytical validation	Rapid detection (~15 min), visual/colorimetric readout, no antibody or labeled probe required (Deng et al., 2026)
OmpC/OmpF	Recombinant-antigen assay	Recombinant-protein ELISA	Preclinical development	Reduces antigen complexity and permits defined antigen composition (Verma, 2009; Neupane et al., 2021)
TolC	Therepeutic/vaccine target	*In vitro* cell-based assay	Target validation/early preclinical research	Bacterial virulence, host cell invasion, and intracellular survival (Hussain et al., 2023; Hussain et al., 2024).
LpxC	Structure-guided inhibition of the Zn²^+^-dependent active site.	Enzyme assays, X-ray crystallography, animal infection models	Clinical development - Phase I completed; discontinued	No validated LpxC diagnostic assay; primarily a therapeutic target (Cole et al., 2011; Krause et al., 2019; Recida Therapeutics, Inc., 2019)
LpxA and LpxD	Dual LpxA/LpxD inhibition and structure-guided inhibitor discovery	SPR, biochemical assays, X-ray crystallography	Hit to lead/early preclinical drug discovery	No validated diagnostic assay (Jenkins et al., 2014)
LpxH	Structure-guided inhibition of UDP-2,3-diacylglucosamine pyrophosphatase	Enzyme assays, X-ray crystallography, MIC assay	Lead optimization/early preclinical drug discovery	No valid diagnostic application (Ennis et al., 2024)
HlyE	Aptamer or antibody binding	ELISA, biosensor, lateral-flow	Early diagnostic development	High specificity for typhoidal strains (Ahmad Najib et al., 2024; Pan et al., 2005; Rm et al., 2020)
Vi- polysachharide	Antibody-mediated recognition of surface-exposed Vi antigen	ELISA, agglutination/serological assays	Clinical, Licenced typhoid vaccines	Anti-Vi antibodies can be detected serologically, although diagnostic sensitivity varies with disease stage and prior vaccination (Snellings et al., 1977; Kulkarni et al., 2024)
Vi polysaccharide–protein conjugates	Vi conjugated to carrier proteins such as CRM197	Glycoconjugate vaccine platform	Clinical implementation/licensed vaccine	preventive platform rather than a diagnostic platform; immunogenicity can be monitored using anti-Vi antibody assays (Micoli et al., 2011).
O-antigen	Antigen detection	Serological assays	Established serological diagnostic application	Widely used but limited specificity (Diwaker et al., 2024; Tran Vu Thieu et al., 2017)
Type IVB pili	Aptamer recognition	Biosensor platforms	Proof-of-concept therapeutic research	Distinguishes typhoidal from non-typhoidal strains (Ahmad Najib et al., 2024; Pan et al., 2005)
CdtB	Recombinant antigen and CdtB-specific antibodies	ELISA, monoclonal antibody	Therapeutic proof-of-concept	Diagnostic: Antigen/antibody detection. Therapeutic: Neutralization of CdtB-mediated cytotoxicity (Raeisi et al., 2022).
PltB	Antibody targeting PltB or the PltA–PltB interface	Monoclonal antibody, ELISA, toxin-binding/neutralization assay	Therapeutic proof-of-concept	Diagnostic: Detection of toxin-associated immune responses. Therapeutic: Prevents toxin assembly, receptor binding and cellular entry. (Ahn et al., 2021)

Advances in protein-targeted diagnostics, notably HlyE aptamers, OMP-focused ELISAs, and toxin-based biosensors, offer promising solutions to long-standing challenges in typhoid detection.

Regardless of the expanding number of protein targets, most anti-virulence and protein-based diagnostic strategies against typhoidal *Salmonella* remain at the preclinical stage. Structural target validation, biochemical inhibition, cellular infection models, animal efficacy studies, pharmacokinetic evaluation, and assessment of resistance emergence should therefore be considered sequential milestones before clinical translation. Among the targets discussed, the Vi antigen has the strongest clinical validation through licensed typhoid conjugate vaccines, whereas LpxC represents an example of structure-guided antibacterial development that progressed to human clinical evaluation but was discontinued because of safety concerns (**Table 6**). Protein-based diagnostic approaches targeting HlyE, FimH, OMPs, and toxin components remain promising but require validation in well-characterized clinical cohorts to establish sensitivity, specificity, disease-stage performance, and discrimination between *S*. Typhi, *S*. Paratyphi A, and non-typhoidal *Salmonella*.

**Table 6:** Protein determinants of typhoidal *Salmonella*: structural features, molecular functions, and translational applications.

Proteins	Structure/domain	Molecular mechanisms	Virulence role	Diagnostic/vaccine/drug potential
SipA PDB: 8VFM, 1Q5Z	Two functional domains: C-terminal actin-binding domain and N-terminal domain.	Directs stabilization and polymerization of F-actin via electrostatic interactions in cooperation with SipC.	Induces host membrane ruffling and macropinocytosis to facilitate bacterial entry and SCV formation.	Anti-virulence target; T3SS inhibitors blocking effector secretion prevent intracellular invasion (Guo et al., 2023).
SipB PDB: 3TUL	Translocator protein with a central hydrophobic region containing two predicted transmembrane helices.	Forms a translocon complex with SipC inserted into host cellular membranes.	Mediates T3SS effector translocation, triggers cell invasion/apoptosis.	Anti-virulence target for small-molecule inhibitors of T3SS translocation platforms (Barta et al., 2012).
SipC PDB: NA	Translocator effector containing a C-terminal SipB-binding domain (residues 340–409).	Cooperates with SipA/SipB to polymerize F-actin and build the translocon pore in host membranes.	Drives localized actin reorganization, membrane ruffling, and internal vacuole formation.	Target for anti-virulence drugs and T3SS secretion inhibitors to block entry into epithelial cells (Myeni et al., 2013).
SipD PDB: 3NZZ	N-terminal region with helices α1 and α2 forming an α-helical hairpin that packs against the coiled coil; helix α3 and a flexible loop (residues 110–132) flank the opposite side	Likely forms a multimeric complex (pentamer/hexamer) at the needle tip, consistent with other T3SS tip proteins leading to membrane ruffling and bacterial uptake	Membrane ruffling and bacterial uptake	serological diagnostic/biomarker candidate because it is a virulence-associated protein and can induce host immune responses (Chatterjee et al., 2011).
SopE PDB: 1GZS	SopE consists of six α-helices arranged into two three-helix bundles, forming a V-shaped architecture	Mimics of host GEFs (Guanine nucleotide exchange factors), thereby activating host Rho GTPases	Cytoskeletal rearrangements	Drug target (Buchwald et al., 2002).
FimH PDB: 9AT9	Tip adhesin with an N-terminal lectin domain (mannose-binding) and a C-terminal pilin domain.	High-affinity binding to mannosylated glycoproteins on host epithelial cells and TLR4 interactions.	Mediates host tissue attachment, initial intestinal epithelial colonization, and macrophage activation.	Target for mannoside-based anti-adhesion drugs, mRNA/subunit vaccines, and diagnostic assays (Lopatto et al., 2024; Roier et al., 2025; Deng et al., 2026).
FimD PDB: 3OHN	Outer membrane usher protein featuring a transmembrane *β*-barrel platform.	Anchors to the outer membrane and orchestrates chaperone-subunit secretion and assembly.	Enables surface assembly and maturation of functional surface fimbriae.	Target for small-molecule usher inhibitors to block fimbrial biogenesis (Kolenda et al., 2019; Uchiya et al., 2019).
OmpC PDB: 5O79	Trimeric *β*-barrel outer membrane porin.	Facilitates passive solute diffusion and forms amyloid-like aggregations.	Promotes nutrient permeability, membrane homeostasis, and host inflammatory responses.	Highly immunogenic adjuvant-like antigen candidate for subunit/multivalent vaccines and diagnostic assays (Singh et al., 1995).
OmpF PDB: 2ZFG	Trimeric *β*-barrel outer membrane porin.	Regulates nutrient permeability and membrane integrity alongside OmpC.	Supports nutrient uptake and survival under host osmotic and inflammatory pressures.	Promising candidate for inclusion in multivalent subunit vaccines to broaden B-cell responses (Pérez-Toledo et al., 2017).
TolC PDB: 7NG9	Outer membrane channel-tunnel protein.	Mediates multidrug efflux pumps and regulates virulence factor secretion.	Essential for antimicrobial resistance, intracellular survival, and suppression of host macrophage responses.	Promising target for antimicrobial drug development, efflux pump inhibitors, and live attenuated vaccines (Hussain et al., 2023; Hussain et al., 2024).
LpxA PDB: 5DEM	Trimeric acyltransferase with a left-handed β-helix core and a small C-terminal α-helical domain	Catalyzes the initial UDP-GlcNAc *O*-acylation step in lipid A biosynthesis.	Essential for outer membrane integrity, endotoxin synthesis, and bacterial viability.	Prime target for novel antibiotics; inhibited by dual-target peptides like RJPXD33 (Smith et al., 2015).
LpxC PDB: 1P42	${\text{Zn}}^{2+}$-dependent metalloamidase with two α/βdomains	Catalyzes the committed deacetylation step in lipid A (endotoxin) biosynthesis.	Essential for Gram-negative cell wall synthesis, outer membrane stability, and survival.	Prominent target for structure-guided antibacterial drugs (e.g., CHIR-090, ACHN-975 and RC-01) (Cole et al., 2011; Krause et al., 2019; Recida Therapeutics, Inc., 2019).
LpxD PDB: 3EH0	Acyltransferase containing a parallel *β*-helix (LβH) domain.	Catalyzes the *N*-acylation step of *R*-3-hydroxymyristoyl groups in lipid A synthesis.	Required for functional lipopolysaccharide assembly and outer membrane permeability barrier.	Drug target for small-molecule antibacterial inhibitors and synthetic peptides (e.g., RJPXD33) (Bartling and Raetz, 2009; Jenkins et al., 2014).
LpxH PDB: 9CD0, PDB: 9CD1	Membrane-associated ${\text{Mn}}^{2+}$-dependent metalloenzyme.	Hydrolyzes UDP-2,3-diacylglucosamine to form lipid X during lipid A assembly.	Essential for maintaining lipid A precursor pools and cell wall integrity.	Candidate for structure-based antibacterial drug discovery targeting its active catalytic pocket (Kwak et al., 2023; Ennis et al., 2024).
Fur PDB: 1MZB	Homodimeric transcriptional regulator with N-terminal DNA-binding and C-terminal dimerization domains.	Undergoes ${\text{Fe}}^{2+}$-induced conformational changes to bind Fur boxes and regulate gene expression.	Regulates iron homeostasis, iron acquisition systems (Fep/Iro), and acid stress responses.	Drug target; disruption of its conserved S2 metal site or peptide aptamers disrupts virulence and iron regulation (Abed et al., 2007; Kang et al., 2024).
Typhoid toxin (PDB: 4K6L/6P4R).	$A_{2}B_{5}$heteroheptamer ( $CdtB+PltA+PltB_{5}$)	CdtBcauses DNA double-strand breaks; PltAacts as ADP-ribosyltransferase; PltBbinds sialoglycans.	Secreted from SCV into blood to destroy immune/host cells and drive systemic typhoid pathogenesis.	Excellent diagnostic biomarker; key candidate for subunit vaccines, neutralizing mAbs (TyTx11, TyTx4), and VHHs (Raeisi et al., 2022; Ahn et al., 2021; Dulal et al., 2021).

## 6 Future perspectives

Future investigations should connect structural biology, AI-assisted drug discovery, single-cell infection models, and protein engineering to accelerate the formulation of protein-targeted anti-virulence approaches against typhoidal *Salmonella.* AlphaFold, virtual screening, X-ray crystallography, and cryo-EM can assist with revealing concealed binding pockets, ligand-binding sites, protein conformations, and protein-protein interactions in significant targets such T3SS proteins, regulatory proteins, and typhoid toxin components. Engineered nanobodies, enhanced vaccine antigens, and outer membrane vesicle platforms may facilitate the creation of specific therapies and vaccinations that safeguard against both *S*. Typhi and *S*. Paratyphi A. Further investigation is required with respect to the underexplored biology of *S*. Paratyphi A, particularly concerning toxin regulation, intracellular survival, and immune evasion mechanisms. Host-directed therapies and clinical trials will be crucial for translating these findings into effective diagnostics, vaccines, and anti-viral treatments.

## 7 Conclusion

Typhoidal *Salmonella* promotes disease through a coordinated set of proteins to adhere to host cells, invade tissue layers, survive inside cells, bypass the immune system, acquire nutrition, and trigger toxin-mediated damage. Comparing *S*. Typhi with *S*. Paratyphi A reveals both common and distinct virulence mechanisms, particularly in their capsule, O-antigen, toxins, and intracellular behavior, which is crucial for developing effective diagnostics, vaccines, and therapies. The available structural evidence supports a transition from descriptive virulence-factor cataloguing toward mechanism-based, structure-guided intervention. Catalytic pockets, receptor-binding sites, conformationally dynamic regions, and protein–protein interfaces represent promising opportunities for anti-virulence drug discovery, toxin neutralization, diagnostic recognition, and rational vaccine design. Future research is needed to characterize the comparatively understudied virulence repertoire of *S.* Paratyphi A, including its unique O-antigen modifications, toxin regulation, and host-adaptation mechanisms. Advances in structural biology, artificial intelligence-assisted drug discovery, and high-throughput screening are expected to accelerate the identification of novel inhibitors against key virulence proteins involved in pathogenesis.

## References

[B1] Abed N. Bickle M. Mari B. Schapira M. Sanjuan-España R. Robbe Sermesant K. . (2007). A comparative analysis of perturbations caused by a gene knock-out, a dominant negative allele, and a set of peptide aptamers. Mol. Cell. Proteomics 6, 2110–2121. doi: 10.1074/mcp.M700105-MCP200 17785351

[B2] Abrahams G. L. Müller P. Hensel M. (2006). Functional dissection of SseF, a type III effector protein involved in positioning the *Salmonella*‐containing vacuole. Traffic 7, 950–965. doi: 10.1111/j.1600-0854.2006.00454.x 16800847

[B3] Adcox H. E. Vasicek E. M. Dwivedi V. Hoang K. V. Turner J. Gunn J. S. (2016). *Salmonella* extracellular matrix components influence biofilm formation and gallbladder colonization. Infect. Immun. 84, 3243–3251. doi: 10.1128/IAI.00532-16 27600501 PMC5067756

[B4] Ahmad Najib M. Winter A. Mustaffa K. M. F. Ong E. B.B. Selvam K. Khalid M. F. . (2024). Isolation and characterization of DNA aptamers against the HlyE antigen of *Salmonella* Typhi. Sci. Rep. 14, 28416. doi: 10.1038/s41598-024-78685-9 39557915 PMC11574307

[B5] Ahn C. Yang Y.-A. Neupane D. P. Nguyen T. Richards A. F. Sim J. H. . (2021). Mechanisms of typhoid toxin neutralization by antibodies targeting glycan receptor binding and nuclease subunits. iScience 24, 102454. doi: 10.1016/j.isci.2021.102454 34113815 PMC8169802

[B6] Althouse C. Patterson S. Fedorka-Cray P. Isaacson R. E. (2003). Type 1 fimbriae of *Salmonella enterica* serovar Typhimurium bind to enterocytes and contribute to colonization of swine *in vivo*. Infect. Immun. 71, 6446–6452. doi: 10.1128/IAI.71.11.6446-6452.2003 14573666 PMC219564

[B7] Amiri M. Golchin M. Jamshidian Mojaver M. Farzin H. Hajizade A. (2025). Enterobactin: A key player in bacterial iron acquisition and virulence and its implications for vaccine development and antimicrobial strategies. Virulence 16, 2563018. doi: 10.1080/21505594.2025.2563018 40966180 PMC12452463

[B8] Andino A. Hanning I. (2015). *Salmonella enterica*: Survival, colonization, and virulence differences among serovars. Sci. World J. 2015, 520179. doi: 10.1155/2015/520179 25664339 PMC4310208

[B9] Arricau N. Hermant D. Waxin H. Ecobichon C. Duffey P. S. Popoff M. Y. (1998). The RcsB-RcsC regulatory system of *Salmonella* Typhi differentially modulates the expression of invasion proteins, flagellin and Vi antigen in response to osmolarity. Mol. Microbiol. 29, 835–850. doi: 10.1046/j.1365-2958.1998.00976.x 9723922

[B10] Barta M. L. Dickenson N. E. Patil M. Keightley A. Wyckoff G. J. Picking W. D. . (2012). The structures of coiled-coil domains from type III secretion system translocators reveal homology to pore-forming toxins. J. Mol. Biol. 417, 395–405. doi: 10.1016/j.jmb.2012.01.026 22321794 PMC3304007

[B11] Bartling C. M. Raetz C. R. H. (2009). Crystal structure and acyl chain selectivity of *Escherichia coli* LpxD, the *N*-acyltransferase of lipid A biosynthesis. Biochemistry 48, 8672–8683. doi: 10.1021/bi901025v 19655786 PMC2748855

[B12] Belousov M. V. Kosolapova A. O. Fayoud H. Sulatsky M. I. Sulatskaya A. I. Romanenko M. N. . (2023). OmpC and OmpF outer membrane proteins of Escherichia coli and *Salmonella enterica* form bona fide amyloids. Int. J. Mol. Sci. 24, 15522. doi: 10.3390/ijms242115522 37958507 PMC10649029

[B13] Bereswill S. Greiner S. van Vliet A. H. M. Waidner B. Fassbinder F. Schiltz E. . (2000). Regulation of ferritin-mediated cytoplasmic iron storage by the ferric uptake regulator homolog (Fur) of Helicobacter pylori. J. Bacteriol. 182, 5948–5953. doi: 10.1128/jb.182.21.5948-5953.2000 11029412 PMC94726

[B14] Berg K. Pedersen H. L. Leiros I. (2020). Biochemical characterization of ferric uptake regulator (Fur) from Aliivibrio salmonicida. Mapping the DNA sequence specificity through binding studies and structural modelling. BioMetals 33, 169–185. doi: 10.1007/s10534-020-00240-6 32648080 PMC7536154

[B15] Bharmoria A. Vaish V. B. (2016). Analysis of attributing characteristics of *Salmonella enterica* serovar Paratyphi A, B and C across India during 6 years, (2010 to 2015). J. Med. Microbiol. Diagnosis 05, 7549–7553. doi: 10.4172/2161-0703.1000220

[B16] Blahut M. Sanchez E. Fisher C. E. Outten F. W. (2020). Fe-S cluster biogenesis by the bacterial Suf pathway. Biochim. Biophys. Acta Mol. Cell. Res. 1867, 118829. doi: 10.1016/j.bbamcr.2020.118829 32822728 PMC7510350

[B17] Bu F. Dee D. R. Liu B. (2024). Structural insight into *Escherichia coli* CsgA amyloid fibril assembly. mBio 15, e00419-24. doi: 10.1128/mbio.00419-24 38501920 PMC11005368

[B18] Buchwald G. Friebel A. Galán J. E. Hardt W.-D. Wittinghofer A. Scheffzek K. (2002). Structural basis for the reversible activation of a Rho protein by the bacterial toxin SopE. EMBO J. 21, 3286–3295. doi: 10.1093/emboj/cdf329 12093730 PMC126081

[B19] Burkinshaw B. J. Prehna G. Worrall L. J. Strynadka N. C. J. (2012). Structure of *Salmonella* effector protein SopB N-terminal domain in complex with host Rho GTPase Cdc42. J. Biol. Chem. 287, 13348–13355. doi: 10.1074/jbc.M111.331330 22362774 PMC3339929

[B20] Buzilă E. R. Dorneanu O. S. Trofin F. Sima C. M. Iancu L. S. (2025). Assessing *Salmonella* Typhi pathogenicity and prevention: The crucial role of vaccination in combating typhoid fever. Int. J. Mol. Sci. 26, 3981. doi: 10.3390/ijms26093981 40362220 PMC12071698

[B21] C W. Barlag B. Gerlach R. G. Deiwick J. Hensel M. (2014). The *Salmonella enterica* giant adhesin SiiE binds to polarized epithelial cells in a lectin-like manner. Cell. Microbiol. 16, 962–975. doi: 10.1111/cmi.12253 24345213

[B22] Cabezudo I. Lobertti C. A. García Véscovi E. Furlan R. L. E. (2022). Effect-directed synthesis of PhoP/PhoQ inhibitors to regulate *Salmonella* virulence. J. Agric. Food. Chem. 70, 6755–6763. doi: 10.1021/acs.jafc.2c01087 35607919

[B23] Cain R. J. Hayward R. D. Koronakis V. (2008). Deciphering interplay between *Salmonella* invasion effectors. PloS Pathog. 4, e1000037. doi: 10.1371/journal.ppat.1000037 18389058 PMC2268969

[B24] Caroff M. Novikov A. (2020). Lipopolysaccharides: Structure, function and bacterial identifications. OCL 27, 31. doi: 10.1051/ocl/2020025

[B25] Cavaliere P. Brier S. Filipenko P. Sizun C. Raynal B. Bonneté F. (2018). The stress sigma factor of RNA polymerase RpoS/σS is a solvent-exposed open molecule in solution. Biochem. J. 475 (1), 341–354. doi: 10.1042/BCJ20170768 29229758

[B26] Caza M. Lépine F. Milot S. Dozois C. M. (2008). Specific roles of the *iroBCDEN* genes in virulence of an avian pathogenic *Escherichia coli* O78 strain and in production of salmochelins. Infect. Immun. 76, 3539–3549. doi: 10.1128/IAI.00455-08 18541653 PMC2493193

[B27] CDC (Typhoid Fever and Paratyphoid Fever) (2024). About typhoid fever and paratyphoid fever. Available online at: https://www.cdc.gov/typhoid-fever/about/index.html.

[B28] Chang S.-J. Hsu Y.-T. Chen Y. Lin Y.-Y. Lara-Tejero M. Galan J. E. (2022). Typhoid toxin sorting and exocytic transport from *Salmonella* Typhi-infected cells. eLife 11, e78561. doi: 10.7554/eLife.78561 35579416 PMC9142146

[B29] Chang S.-J. Jin S. C. Jiao X. Galán J. E. (2019). Unique features in the intracellular transport of typhoid toxin revealed by a genome-wide screen. PloS Pathog. 15, e1007704. doi: 10.1371/journal.ppat.1007704 30951565 PMC6469816

[B30] Chatterjee S. Zhong D. Nordhues B. A. Battaile K. P. Lovell S. De Guzman R. N. (2011). The crystal structures of the *Salmonella* type III secretion system tip protein SipD in complex with deoxycholate and chenodeoxycholate. Protein Sci. 20, 75–86. doi: 10.1002/pro.537 21031487 PMC3047063

[B31] Choby J. E. Mike L. A. Mashruwala A. A. Dutter B. F. Dunman P. M. Sulikowski G. A. . (2016). A small-molecule inhibitor of iron-sulfur cluster assembly uncovers a link between virulence regulation and metabolism in Staphylococcus aureus. Cell Chem. Biol. 23, 1351–1361. doi: 10.1016/j.chembiol.2016.09.012 27773628 PMC5117899

[B32] Choi E. Groisman E. A. Shin D. (2009). Activated by different signals, the PhoP/PhoQ two-component system differentially regulates metal uptake. J. Bacteriol. 191, 7174–7181. doi: 10.1128/JB.00958-09 19801407 PMC2786564

[B33] Choi J. Ryu S. (2019). Regulation of iron uptake by fine-tuning the iron responsiveness of the iron sensor Fur. Appl. Environ. Microbiol. 85, e03026-18. doi: 10.1128/AEM.03026-18 30824449 PMC6495754

[B34] Cole K. E. Gattis S. G. Angell H. D. Fierke C. A. Christianson D. W. (2011). Structure of the metal-dependent deacetylase LpxC from *Yersinia enterocolitica* complexed with the potent inhibitor CHIR-090. Biochemistry 50, 258–265. doi: 10.1021/bi101622a 21171638 PMC3070812

[B35] Costa L. F. Mol J. P. S. Silva A. P. C. Macêdo A. A. Silva T. M.A. Alves G. E.S. . (2016). Iron acquisition pathways and colonization of the inflamed intestine by *Salmonella enterica* serovar Typhimurium. Int. J. Med. Microbiol. 306, 604–610. doi: 10.1016/j.ijmm.2016.10.004 27760693 PMC5140723

[B36] Crouch M.-L. V. Castor M. Karlinsey J. E. Kalhorn T. Fang F. C. (2008). Biosynthesis and IroC-dependent export of the siderophore salmochelin are essential for virulence of *Salmonella enterica* serovar Typhimurium. Mol. Microbiol. 67, 971–983. doi: 10.1111/j.1365-2958.2007.06089.x 18194158

[B37] Cunrath O. Palmer J. D. (2021). An overview of *Salmonella enterica* metal homeostasis pathways during infection. microLife 2, uqab001. doi: 10.1093/femsml/uqab001 34250489 PMC8264917

[B38] De Castro C. Parrilli M. Holst O. Molinaro A. (2010). “ Chapter five - Microbe-associated molecular patterns in innate immunity: Extraction and chemical analysis of gram-negative bacterial lipopolysaccharides,” in Glycobiology, vol. 480 . Ed. Fukuda M. (Amsterdam, Netherlands: Elsevier). doi: 10.1016/S0076-6879(10)80005-9 20816206

[B39] Del Bel Belluz L. Guidi R. Pateras I. S. Levi L. Mihaljevic B. Rouf S. F. . (2016). The typhoid toxin promotes host survival and the establishment of a persistent asymptomatic infection. PloS Pathog. 12, e1005528. doi: 10.1371/journal.ppat.1005528 27055274 PMC4824513

[B40] Deng Z. Tian J. Zhao L. Liu X. Yin X. Wang J. . (2026). Self-oxygenating nanozyme-based biosensor for rapid detection of *Salmonella* Typhimurium. Biosens. Bioelectron. 293, 118162. doi: 10.1016/j.bios.2025.118162 41172908

[B41] Diacovich L. Dumont A. Lafitte D. Soprano E. Guilhon A.-A. Bignon C. . (2009). Interaction between the SifA virulence factor and its host target SKIP is essential for *Salmonella* pathogenesis. J. Biol. Chem. 284, 33151–33160. doi: 10.1074/jbc.M109.034975 19801640 PMC2785157

[B42] Ding H. (2025). Iron-sulfur cluster biogenesis and regulation of intracellular iron homeostasis in *Escherichia coli*. Metallomics: Integrated Biometal Sci. 17, mfaf040. doi: 10.1093/mtomcs/mfaf040 41263481 PMC12683245

[B43] Diwaker A. Tiwari A. Jain S. Rupali K. A. Ram J. Singh S. . (2024). Enteric fever and the diagnostic tools: Defining the accuracy. Front. Bacteriology 3. doi: 10.3389/fbrio.2024.1332180

[B44] Duguid J. P. Smith I. W. Dempster G. Edmunds P. N. (1955). Non-flagellar filamentous appendages (fimbriae) and haemagglutinating activity in bacterium coli. J. Pathol. Bacteriol. 70, 335–348. doi: 10.1002/path.1700700210 13295908

[B45] Dulal H. P. Vance D. J. Neupane D. P. Chen X. Tremblay J. M. Shoemaker C. B. . (2021). Neutralization of typhoid toxin by alpaca-derived, single-domain antibodies targeting the PltB and CdtB subunits. Infect. Immun. 90, ahead of print. doi: 10.1128/iai.00515-21 34898253 PMC8852740

[B46] Dyson Z. A. Klemm E. J. Palmer S. Dougan G. (2019). Antibiotic resistance and typhoid. Clin. Infect. Diseases: An. Off. Publ. Infect. Dis. Soc. America 68, S165–S170. doi: 10.1093/cid/ciy1111 30845331 PMC6405283

[B47] Ennis A. F. Cochrane C. S. Dome P. A. Jeong P. Yu J. Lee H. . (2024). Design and evaluation of pyridinyl sulfonyl piperazine LpxH inhibitors with potent antibiotic activity against Enterobacterales. JACS Au 4, 4383–4393. doi: 10.1021/jacsau.4c00731 39610720 PMC11600146

[B48] Esquilin-Lebron K. Dubrac S. Barras F. Boyd J. M. (2021). Bacterial approaches for assembling iron-sulfur proteins. mBio 12, e0242521. doi: 10.1128/mBio.02425-21 34781750 PMC8593673

[B49] Fass E. Groisman E. A. (2009). Control of *Salmonella* pathogenicity island-2 gene expression. Curr. Opin. Microbiol. 12, 199–204. doi: 10.1016/j.mib.2009.01.004 19264535 PMC2805070

[B50] Fernandez-Beros M. E. Gonzalez C. McIntosh M. A. Cabello F. C. (1989). Immune response to the iron-deprivation-induced proteins of *Salmonella* Typhi in typhoid fever. Infect. Immun. 57, 1271–1275. doi: 10.1128/iai.57.4.1271-1275.1989 2522420 PMC313260

[B51] Foster J. W. Hall H. K. (1992). Effect of *Salmonella* Typhimurium ferric uptake regulator (Fur) mutations on iron- and pH-regulated protein synthesis. J. Bacteriol. 174, 4317–4323. doi: 10.1128/jb.174.13.4317-4323.1992 1624426 PMC206215

[B52] Fowler C. C. Stack G. Jiao X. Lara-Tejero M. Galán J. E. (2019). Alternate subunit assembly diversifies the function of a bacterial toxin. Nat. Commun. 10, 3684. doi: 10.1038/s41467-019-11592-0 31417089 PMC6695444

[B53] Friebel A. Ilchmann H. Aepfelbacher M. Ehrbar K. Machleidt W. Hardt W. D. (2001). SopE and SopE2 from *Salmonella* Typhimurium activate different sets of RhoGTPases of the host cell. J. Biol. Chem. 276, 34035–34040. doi: 10.1074/jbc.M100609200 11440999

[B54] Fuentes J. A. Villagra N. Castillo-Ruiz M. Mora G. C. (2008). The *Salmonella* Typhi hlyE gene plays a role in invasion of cultured epithelial cells and its functional transfer to S. Typhimurium promotes deep organ infection in mice. Res. Microbiol. 159, 279–287. doi: 10.1016/j.resmic.2008.02.006 18434098

[B55] Furuya T. Shapiro A. B. Comita-Prevoir J. Kuenstner E. J. Zhang J. Ribe S. D. . (2020). N-hydroxyformamide LpxC inhibitors, their *in vivo* efficacy in a mouse Escherichia coli infection model, and their safety in a rat hemodynamic assay. Bioorg. Med. Chem. 28, 115826. doi: 10.1016/j.bmc.2020.115826 33160146

[B56] Galán J. E. (2026). Typhoid toxin: Reframing enteric fever. Microbiol. Mol. Biol. Reviews: MMBR x, e0000825. doi: 10.1128/mmbr.00008-25 42041253 PMC13296355

[B57] Gal-Mor O. Boyle E. C. Grassl G. A. (2014). Same species, different diseases: How and why typhoidal and non-typhoidal *Salmonella enterica* serovars differ. Front. Microbiol. 5, 391. doi: 10.3389/fmicb.2014.00391 25136336 PMC4120697

[B58] Gasperini G. Alfini R. Arato V. Mancini F. Aruta M. G. Kanvatirth P. . (2021). *Salmonella* Paratyphi A outer membrane vesicles displaying Vi polysaccharide as a multivalent vaccine against enteric fever. Infect. Immun. 89, e00699-20. doi: 10.1128/IAI.00699-20 33318138 PMC8090967

[B59] Ghosh S. Chakraborty K. Nagaraja T. Basak S. Koley H. Dutta S. . (2011). An adhesion protein of *Salmonella enterica* serovar Typhi is required for pathogenesis and potential target for vaccine development. PNAS 108, 3348–3353. doi: 10.1073/pnas.1016180108 21300870 PMC3044360

[B60] Glynn J. R. Palmer S. R. (1992). Incubation period, severity of disease, and infecting dose: Evidence from a *Salmonella* outbreak. Am. J. Epidemiol. 136, 1369–1377. doi: 10.1093/oxfordjournals.aje.a116449 1488963

[B61] González J. F. Laipply B. Sadowski V. A. Price M. Gunn J. S. (2024). Functional role of the biofilm regulator CsgD in *Salmonella enterica* Sv. Typhi. Front. Cell. Infect. Microbiol. 14, 1478488. doi: 10.3389/fcimb.2024.1478488 39720794 PMC11668344

[B62] Gordon M. A. (2008). *Salmonella* infections in immunocompromised adults. J. Infection 56, 413–422. doi: 10.1016/j.jinf.2008.03.012 18474400

[B63] Grizot S. Salem M. Vongsouthi V. Durand L. Moreau F. Dohi H. . (2006). Structure of the Escherichia coli heptosyltransferase WaaC: Binary complexes with ADP and ADP-2-deoxy-2-fluoro heptose. J. Mol. Biol. 363, 383–394. doi: 10.1016/j.jmb.2006.07.057 16963083

[B64] Guerra F. E. Karlinsey J. E. Libby S. J. Fang F. C. (2025). Evasion of serum antibodies and complement by *Salmonella* Typhi and Paratyphi A. PloS Pathog. 21, e1012917. doi: 10.1371/journal.ppat.1012917 40315236 PMC12068720

[B65] Gunn J. S. Bakaletz L. O. Wozniak D. J. (2016). What’s on the outside matters: The role of the extracellular polymeric substance of Gram-negative biofilms in evading host immunity and as a target for therapeutic intervention. J. Biol. Chem. 291, 12538–12546. doi: 10.1074/jbc.R115.707547 27129225 PMC4933457

[B66] Guo E. Z. Chou S. Z. Lara-Tejero M. Galán J. E. (2023). Cryo-EM structure of the bacterial effector protein SipA bound to F-actin reveals a unique mechanism for filament stabilization. Microbiology. doi: 10.1101/2023.12.21.572903 38187563 PMC10769390

[B67] Halsey T. A. Vazquez-Torres A. Gravdahl D. J. Fang F. C. Libby S. J. (2004). The ferritin-like Dps protein is required for *Salmonella enterica* serovar Typhimurium oxidative stress resistance and virulence. Infect. Immun. 72, 1155–1158. doi: 10.1128/IAI.72.2.1155-1158.2004 14742565 PMC321587

[B68] Hantke K. Nicholson G. Rabsch W. Winkelmann G. (2003). Salmochelins, siderophores of *Salmonella enterica* and uropathogenic Escherichia coli strains, are recognized by the outer membrane receptor IroN. Proc. Natl. Acad. Sci. 100, 3677–3682. doi: 10.1073/pnas.0737682100 12655053 PMC152981

[B69] Hassanin A. Lories B. Steenackers H. P. (2025). *Salmonella* stress response systems as targets for anti-virulence strategies. BMC Microbiol. 25, 378. doi: 10.1186/s12866-025-04003-6 40596824 PMC12217294

[B70] Henry T. Couillault C. Rockenfeller P. Boucrot E. Dumont A. Schroeder N. . (2006). The *Salmonella* effector protein PipB2 is a linker for kinesin-1. Proc. Natl. Acad. Sci. 103, 13497–13502. doi: 10.1073/pnas.0605443103 16938850 PMC1569191

[B71] Hiyoshi H. Tiffany C. R. Bronner D. N. Bäumler A. J. (2018). Typhoidal *Salmonella* serovars: Ecological opportunity and the evolution of a new pathovar. FEMS Microbiol. Rev. 42, 527–541. doi: 10.1093/femsre/fuy024 29790924

[B72] Hoare A. Bittner M. Carter J. Alvarez S. Zaldívar M. Bravo D. . (2006). The outer core lipopolysaccharide of *Salmonella enterica* serovar Typhi is required for bacterial entry into epithelial cells. Infect. Immun. 74, 1555–1564. doi: 10.1128/IAI.74.3.1555-1564.2006 16495526 PMC1418631

[B73] Hummels K. R. (2025). The regulation of lipid A biosynthesis. J. Biol. Chem. 301, 110556. doi: 10.1016/j.jbc.2025.110556 40752575 PMC12444477

[B74] Hussain A. Ong E. B. B. Balaram P. Ismail A. Kien P. K. (2023). Deletion of *Salmonella enterica* serovar Typhi tolC reduces bacterial adhesion and invasion toward host cells. Front. Microbiol. 14, 1301478. doi: 10.3389/fmicb.2023.1301478 38029101 PMC10655110

[B75] Hussain A. Ong E. B. B. Balaram P. Ismail A. Kien P. K. (2024). TolC facilitates the intracellular survival and immunomodulation of *Salmonella* Typhi in human host cells. Virulence 15, 2395831. doi: 10.1080/21505594.2024.2395831 39185619 PMC11385165

[B76] Ishii E. Eguchi Y. (2021). Diversity in sensing and signaling of bacterial sensor histidine kinases. Biomolecules 11, 1524. doi: 10.3390/biom11101524 34680156 PMC8534201

[B77] Islam S. T. Lam J. S. (2014). Synthesis of bacterial polysaccharides via the Wzx/Wzy-dependent pathway. Can. J. Microbiol. 60, 697–716. doi: 10.1139/cjm-2014-0595 25358682

[B78] Islam S. T. Taylor V. L. Qi M. Lam J. S. (2010). Membrane topology mapping of the O-antigen flippase (Wzx), polymerase (Wzy), and ligase (WaaL) from Pseudomonas aeruginosa PAO1 reveals novel domain architectures. mBio 1, e00189-10. doi: 10.1128/mBio.00189-10 20824106 PMC2932511

[B79] J Barton A. Hill J. Blohmke C. J. Pollard A. J. (2021). Host restriction, pathogenesis and chronic carriage of typhoidal *Salmonella*. FEMS Microbiol. Rev. 45, fuab014. doi: 10.1093/femsre/fuab014 33733659 PMC8498562

[B80] Jenkins R. J. Heslip K. A. Meagher J. L. Stuckey J. A. Dotson G. D. (2014). Structural basis for the recognition of peptide RJPXD33 by acyltransferases in lipid A biosynthesis*. J. Biol. Chem. 289, 15527–15535. doi: 10.1074/jbc.M114.564278 24742680 PMC4140908

[B81] Jia H. Song N. Ma Y. Zhang F. Yue Y. Wang W. . (2022). *Salmonella* facilitates iron acquisition through UMPylation of ferric uptake regulator. mBio 13, e00207-22. doi: 10.1128/mbio.00207-22 35532216 PMC9239237

[B82] Jiao X. Smith S. Stack G. Liang Q. Bradley A. Kellam P. . (2020). Generation and characterization of typhoid toxin-neutralizing human monoclonal antibodies. Infect. Immun. 88, ahead of print. doi: 10.1128/iai.00292-20 32661121 PMC7504945

[B83] Johnson R. Byrne A. Berger C. N. Klemm E. Crepin V. F. Dougan G. . (2017). The type III secretion system effector SptP of *Salmonella enterica* serovar Typhi. J. Bacteriol. 199, e00647-16. doi: 10.1128/JB.00647-16 27920299 PMC5287405

[B84] Johnson R. Mylona E. Frankel G. (2018a). Typhoidal *Salmonella*: Distinctive virulence factors and pathogenesis. Cell. Microbiol. 20, e12939. doi: 10.1111/cmi.12939 30030897

[B85] Johnson R. Ravenhall M. Pickard D. Dougan G. Byrne A. Frankel G. (2018b). Comparison of *Salmonella enterica* serovars Typhi and Typhimurium reveals typhoidal serovar-specific responses to bile. Infect. Immun. 86, e00490-17. doi: 10.1128/IAI.00490-17 29229736 PMC5820949

[B86] Kang S.-M. Kang H.-S. Chung W.-H. Kang K.-T. Kim D.-H. (2024). Structural perspectives on metal dependent roles of ferric uptake regulator (Fur). Biomolecules 14, 981. doi: 10.3390/biom14080981 39199369 PMC11353095

[B87] Karas V. O. Westerlaken I. Meyer A. S. (2015). The DNA-binding protein from starved cells (Dps) utilizes dual functions to defend cells against multiple stresses. J. Bacteriol. 197, 3206–3215. doi: 10.1128/JB.00475-15 26216848 PMC4560292

[B88] Karthikeyan D. Kumar S. Jayaprakash N. S. (2025). Pharmacophore-based approach for the identification of prospective UDP-2,3-diacylglucosamine hydrolase (LpxH) inhibitor from natural product database against *Salmonella* Typhi. Chem. Phys. Impact 10, 100824. doi: 10.1016/j.chphi.2025.10082442574925

[B89] Kasberg W. Luong P. Hanna M. G. Minushkin K. Tsao A. Shankar R. . (2023). The Sar1 GTPase is dispensable for COPII-dependent cargo export from the ER. Cell Rep. 42, 112635. doi: 10.1016/j.celrep.2023.112635 37300835 PMC10592460

[B90] Kaur J. Jain S. (2012). Vi antigen of *Salmonella enetrica* serovar Typhi — biosynthesis, regulation and its use as vaccine candidate. Open Life Sci. 7, 825–838. doi: 10.2478/s11535-012-0082-8

[B91] Khare G. Nangpal P. Tyagi A. K. (2017). Differential roles of iron storage proteins in maintaining the iron homeostasis in Mycobacterium tuberculosis. PloS One 12, e0169545. doi: 10.1371/journal.pone.0169545 28060867 PMC5218490

[B92] Kintz E. Heiss C. Black I. Donohue N. Brown N. Davies M. R. . (2017). *Salmonella enterica* serovar Typhi lipopolysaccharide O-antigen modification impact on serum resistance and antibody recognition. Infect. Immun. 85, e01021-16. doi: 10.1128/IAI.01021-16 28167670 PMC5364305

[B93] Kline K. A. Fälker S. Dahlberg S. Normark S. Henriques-Normark B. (2009). Bacterial adhesins in host-microbe interactions. Cell. Host Microbe 5, 580–592. doi: 10.1016/j.chom.2009.05.011 19527885

[B94] Kolenda R. Ugorski M. Grzymajlo K. (2019). Everything you always wanted to know about *Salmonella* type 1 fimbriae, but were afraid to ask. Front. Microbiol. 10, 1017. doi: 10.3389/fmicb.2019.01017 31139165 PMC6527747

[B95] Krause K. M. Haglund C. M. Hebner C. Serio A. W. Lee G. Nieto V. . (2019). Potent LpxC inhibitors with *in vitro* activity against multidrug-resistant Pseudomonas aeruginosa. Antimicrob. Agents Chemother. 63, e00977-19. doi: 10.1128/AAC.00977-19 31451507 PMC6811409

[B96] Kuehn R. Rahden P. Syed Hussain H. Karkey A. Qamar F. N. Rupali P. . (2025). Enteric (typhoid and paratyphoid) fever. Lancet 406, 1283–1294. doi: 10.1016/S0140-6736(25)01335-2 40914181

[B97] Kulkarni P. S. Potey A. V. Bharati S. Kunhihitlu A. Narasimha B. Yallapa S. . (2024). The safety and immunogenicity of a bivalent conjugate vaccine against *Salmonella enterica* Typhi and Paratyphi A in healthy Indian adults: A phase 1, randomised, active-controlled, double-blind trial. Lancet 403, 1554–1562. doi: 10.1016/S0140-6736(24)00249-6 38555928

[B98] Kurabayashi K. Hirakawa Y. Tanimoto K. Tomita H. Hirakawa H. (2014). Role of the CpxAR two-component signal transduction system in control of fosfomycin resistance and carbon substrate uptake. J. Bacteriol. 196, 248–256. doi: 10.1128/JB.01151-13 24163343 PMC3911262

[B99] Kwak S. Cochrane C. S. Cho J. Dome P. A. Ennis A. F. Kim J. H. . (2023). Development of LpxH inhibitors chelating the active site dimanganese metal cluster of LpxH. ChemMedChem 18, e202300023. doi: 10.1002/cmdc.202300023 37014664 PMC10239344

[B100] Lara-Tejero M. Galán J. E. (2001). CdtA, CdtB, and CdtC form a tripartite complex that is required for cytolethal distending toxin activity. Infect. Immun. 69, 4358–4365. doi: 10.1128/IAI.69.7.4358-4365.2001 11401974 PMC98507

[B101] Latasa C. Roux A. Toledo-Arana A. Ghigo J.-M. Gamazo C. Penadés J. R. . (2005). BapA, a large secreted protein required for biofilm formation and host colonization of *Salmonella enterica* serovar Enteritidis. Mol. Microbiol. 58, 1322–1339. doi: 10.1111/j.1365-2958.2005.04907.x 16313619

[B102] Lee H. Hsu F. F. Turk J. Groisman E. A. (2004). The PmrA-regulated pmrC gene mediates phosphoethanolamine modification of lipid A and polymyxin resistance in *Salmonella enterica*. J. Bacteriol. 186, 4124–4133. doi: 10.1128/JB.186.13.4124-4133.2004 15205413 PMC421605

[B103] Lee S. Yang Y. A. Milano S. K. Nguyen T. Ahn C. Sim J. H. . (2020). *Salmonella* typhoid toxin PltB subunit and its non-typhoidal *Salmonella* ortholog confer differential host adaptation and virulence. Cell. Host Microbe 27, 937–949.e6. doi: 10.1016/j.chom.2020.04.005 32396840 PMC7292776

[B104] Li Q. (2022). Mechanisms for the invasion and dissemination of *Salmonella*. Can. J. Infect. Dis. Med. Microbiol. = J. Canadien Des. Maladies Infectieuses La Microbiologie Médicale 2022, 2655801. doi: 10.1155/2022/2655801 35722038 PMC9203224

[B105] Lim D. Kim K. Song M. Jeong J.-H. Chang J. H. Kim S. R. . (2020). Transcriptional regulation of salmochelin glucosyltransferase by Fur in *Salmonella*. Biochem. Biophys. Res. Commun. 529, 70–76. doi: 10.1016/j.bbrc.2020.06.009 32560822

[B106] Lin H. Fischbach M. A. Liu D. R. Walsh C. T. (2005). *In vitro* characterization of salmochelin and enterobactin trilactone hydrolases IroD, IroE, and Fes. J. Am. Chem. Soc 127, 11075–11084. doi: 10.1021/ja0522027 16076215 PMC2536649

[B107] Lopatto E. D. B. Pinkner J. S. Sanick D. A. Potter R. F. Liu L. X. Bazán Villicaña J. . (2024). Conformational ensembles in Klebsiella pneumoniae FimH impact uropathogenesis. Proc. Natl. Acad. Sci. 121, e2409655121. doi: 10.1073/pnas.2409655121 39288182 PMC11441496

[B108] Lou L. Zhang P. Piao R. Wang Y. (2019). *Salmonella* pathogenicity island 1 (SPI-1) and its complex regulatory network. Front. Cell. Infect. Microbiol. 9, 270. doi: 10.3389/fcimb.2019.00270 31428589 PMC6689963

[B109] Malik-Kale P. Jolly C. E. Lathrop S. Winfree S. Luterbach C. Steele-Mortimer O. (2011). *Salmonella* - at home in the host cell. Front. Microbiol. 2, 125. doi: 10.3389/fmicb.2011.00125 21687432 PMC3109617

[B110] Mao M. He L. Yan Q. (2025). An updated overview on the bacterial PhoP/PhoQ two-component signal transduction system. Front. Cell. Infect. Microbiol. 15, 1509037. doi: 10.3389/fcimb.2025.1509037 39958932 PMC11825808

[B111] Marolda C. L. Tatar L. D. Alaimo C. Aebi M. Valvano M. A. (2006). Interplay of the Wzx translocase and the corresponding polymerase and chain lengthregulator proteins in the translocation and periplasmic assembly of lipopolysaccharide O antigen. J. Bacteriol. 188, 5124–5135. doi: 10.1128/JB.00461-06 16816184 PMC1539953

[B112] Marondedze E. F. Govender P. P. (2022). Exploiting the glycan receptor-binding site of PltB subunit in *Salmonella* Typhi toxin for novel inhibitors: An in-silico approach. J. Mol. Graphics Modell. 111, 108082. doi: 10.1016/j.jmgm.2021.108082 34837784

[B113] Mastroeni P. Rossi O. (2020). Antibodies and protection in systemic *Salmonella* infections: Do we still have more questions than answers? Infect. Immun. 88, e00219-20. doi: 10.1128/IAI.00219-20 32601109 PMC7504966

[B114] Mey A. R. Gómez-Garzón C. Payne S. M. (2021). Iron transport and metabolism in *Escherichia*, *Shigella*, and *Salmonella*. EcoSal Plus 9, eESP00342020. doi: 10.1128/ecosalplus.ESP-0034-2020 34910574 PMC8865473

[B115] Micoli F. Rondini S. Gavini M. Lanzilao L. Medaglini D. Saul A. . (2012). O:2-CRM(197) conjugates against *Salmonella* Paratyphi A. PloS One 7, e47039. doi: 10.1371/journal.pone.0047039 23144798 PMC3492368

[B116] Micoli F. Rondini S. Pisoni I. Proietti D. Berti F. Costantino P. . (2011). Vi-CRM197 as a new conjugate vaccine against *Salmonella* Typhi. Vaccine 29, 712–720. doi: 10.1016/j.vaccine.2010.11.022 21115057 PMC4163788

[B117] Moll I. Leitsch D. Steinhauser T. Bläsi U. (2003). RNA chaperone activity of the Sm‐like Hfq protein. EMBO Rep. 4, 284–289. doi: 10.1038/sj.embor.embor772 12634847 PMC1315900

[B118] Motz R. N. Anderson J. K. Nolan E. M. (2025). Re-evaluation of the C -glucosyltransferase IroB illuminates its ability to C -glucosylate non-native triscatecholate enterobactin mimics. Biochemistry 64, 224–237. doi: 10.1021/acs.biochem.4c00581 39718537 PMC12228543

[B119] Myeni S. K. Wang L. Zhou D. (2013). SipB-SipC complex is essential for translocon formation. PloS One 8, e60499. doi: 10.1371/journal.pone.0060499 23544147 PMC3609803

[B120] Mylona E. Sanchez-Garrido J. Nguyen Hoang Thu T. Dongol S. Karkey A. Baker S. . (2021). Very long O-antigen chains of *Salmonella* Paratyphi A inhibit inflammasome activation and pyroptotic cell death. Cell. Microbiol. 23, e13306. doi: 10.1111/cmi.13306 33355403 PMC8609438

[B121] Myriam P. Paillavil B. Rivera-Araya J. Claudia M. V. Omar O. Renato C. . (2022). Insights into systems for iron-sulfur cluster biosynthesis in acidophilic microorganisms. J. Microbiol. Biotechnol. 32, 1110–1119. doi: 10.4014/jmb.2206.06045 36039043 PMC9628965

[B122] Nagy T. A. Moreland S. M. Andrews-Polymenis H. Detweiler C. S. (2013). The ferric enterobactin transporter Fep is required for persistent *Salmonella enterica* serovar Typhimurium infection. Infect. Immun. 81, 4063–4070. doi: 10.1128/iai.00412-13 23959718 PMC3811836

[B123] Neupane D. P. Dulal H. P. Song J. (2021). Enteric fever diagnosis: Current challenges and future directions. Pathogens 10, 410. doi: 10.3390/pathogens10040410 33915749 PMC8065732

[B124] Nevermann J. Silva A. Otero C. Oyarzún D. P. Barrera B. Gil F. . (2019). Identification of genes involved in biogenesis of outer membrane vesicles (OMVs) in *Salmonella enterica* serovar Typhi. Front. Microbiol. 10. doi: 10.3389/fmicb.2019.00104 30778340 PMC6369716

[B125] Nguyen Q. C. Everest P. Tran T. K. House H. Murch S. Parry C. . (2004). A clinical, microbiological, and pathological study of intestinal perforation associated with typhoid fever. Clin. Infect. Diseases: An. Off. Publ. Infect. Dis. Soc. America 39, 61–67. doi: 10.1086/421555 15206054

[B126] Nickerson C. A. Curtiss R. (1997). Role of sigma factor RpoS in initial stages of *Salmonella* Typhimurium infection. Infect. Immun. 65, 1814–1823. doi: 10.1128/iai.65.5.1814-1823.1997 9125566 PMC175223

[B127] Olsen S. J. Pruckler J. Bibb W. Nguyen T. M. T. Tran M. T. Nguyen T. M. . (2004). Evaluation of rapid diagnostic tests for typhoid fever. J. Clin. Microbiol. 42, 1885–1889. doi: 10.1128/JCM.42.5.1885-1889.2004 15131144 PMC404619

[B128] Orban K. Finkel S. E. (2022). Dps is a universally conserved dual-action DNA-binding and ferritin protein. J. Bacteriol. 204, e00036-22. doi: 10.1128/jb.00036-22 35380871 PMC9112962

[B129] Outten F. W. (2015). Recent advances in the Suf Fe-S cluster biogenesis pathway: Beyond the Proteobacteria. Biochim. Biophys. Acta 1853, 1464–1469. doi: 10.1016/j.bbamcr.2014.11.001 25447545 PMC4390423

[B130] Pan Q. Zhang X.-L. Wu H.-Y. He P.-W. Wang F. Zhang M.-S. . (2005). Aptamers that preferentially bind type IVB pili and inhibit human monocytic-cell invasion by *Salmonella enterica* serovar Typhi. Antimicrob. Agents Chemother. 49, 4052–4060. doi: 10.1128/AAC.49.10.4052-4060.2005 16189080 PMC1251553

[B131] Parkhill J. Dougan G. James K. D. Thomson N. R. Pickard D. Wain J. . (2001). Complete genome sequence of a multiple drug resistant *Salmonella enterica* serovar Typhi CT18. Nature 413, 848–852. doi: 10.1038/35101607 11677608

[B132] Pérez-Toledo M. Valero-Pacheco N. Pastelin-Palacios R. Gil-Cruz C. Perez-Shibayama C. Moreno-Eutimio M. A. . (2017). *Salmonella* Typhi porins OmpC and OmpF are potent adjuvants for T-dependent and T-independent antigens. Front. Immunol. 8. doi: 10.3389/fimmu.2017.00230 28337196 PMC5344031

[B133] Pinto M. Durante S. Carducci M. Massai L. Alfini R. Mylona E. . (2025). The *Salmonella* Paratyphi A O-antigen glycoconjugate vaccine is able to induce antibodies with bactericidal activity against a panel of clinical isolates. Vaccines. 13, 122. doi: 10.3390/vaccines13020122 40006669 PMC11860196

[B134] Piovani D. Figlioli G. Nikolopoulos G. K. Bonovas S. (2024). The global burden of enteric fever 2017-2021: A systematic analysis from the Global Burden of Disease Study 2021. EClinicalMedicine 77, 102883. doi: 10.1016/j.eclinm.2024.102883 39469533 PMC11513656

[B135] Piscatelli H. L. Li M. Zhou D. (2016). Dual 4- and 5-phosphatase activities regulate SopB-dependent phosphoinositide dynamics to promote bacterial entry: Regulation of SopB-dependent phosphoinositide dynamics. Cell. Microbiol. 18, 705–719. doi: 10.1111/cmi.12542 26537021

[B136] Pohl E. Haller J. C. Mijovilovich A. Meyer‐Klaucke W. Garman E. Vasil M. L. (2003). Architecture of a protein central to iron homeostasis: Crystal structure and spectroscopic analysis of the ferric uptake regulator. Mol. Microbiol. 47, 903–915. doi: 10.1046/j.1365-2958.2003.03337.x 12581348

[B137] Pons B. Vignard J. Mirey G. (2019). Cytolethal distending toxin subunit B: A review of structure–function relationship. Toxins 11, 595. doi: 10.3390/toxins11100595 31614800 PMC6832162

[B138] Rabsch W. Voigt W. Reissbrodt R. Tsolis R. M. Bäumler A. J. (1999). *Salmonella* Typhimurium IroN and FepA proteins mediate uptake of enterobactin but differ in their specificity for other siderophores. J. Bacteriol. 181, 3610–3612. doi: 10.1128/jb.181.11.3610-3612.1999 10348879 PMC93834

[B139] Raeisi H. Azimirad M. Nabavi-Rad A. Asadzadeh Aghdaei H. Yadegar A. Zali M. R. (2022). Application of recombinant antibodies for treatment of Clostridioides difficile infection: Current status and future perspective. Front. Immunol. 13, 972930. doi: 10.3389/fimmu.2022.972930 36081500 PMC9445313

[B140] Raetz C. R. H. Reynolds C. M. Trent M. S. Bishop R. E. (2007). Lipid a modification systems in gram-negative bacteria. Annu. Rev. Biochem. 76, 295–329. doi: 10.1146/annurev.biochem.76.010307.145803 17362200 PMC2569861

[B141] Ramos-Morales F. (2012). Impact of *Salmonella enterica* type III secretion system effectors on the eukaryotic host cell. Int. Scholarly Res. Notices 2012, 787934. doi: 10.5402/2012/78793442642048

[B142] Recida Therapeutics, Inc (2019). A Phase 1, Double-Blind, Dose Escalation Study to Evaluate the Safety, Tolerability, and Pharmacokinetics of RC-01 for Injection in Healthy Adult Subjects. Available online at: https://clinicaltrials.gov/study/NCT03832517 (Accessed September 01, 2026).

[B143] Rehman T. Yin L. Latif M. B. Chen J. Wang K. Geng Y. . (2019). Adhesive mechanism of different *Salmonella* fimbrial adhesins. Microb. Pathogen. 137, 103748. doi: 10.1016/j.micpath.2019.103748 31521802

[B144] Rivera M. (2023). Mobilization of iron stored in bacterioferritin, a new target for perturbing iron homeostasis and developing antibacterial and antibiofilm molecules. J. Inorg. Biochem. 247, 112306. doi: 10.1016/j.jinorgbio.2023.112306 37451083 PMC11642381

[B145] Rm R. Maroli N. Achuth J. Kolandaivel P. Kadirvelu K. (2020). Highly adaptable and sensitive FRET-based aptamer assay for the detection of *Salmonella* Paratyphi A. Spectrochimica Acta Part. A. Mol. Biomolecular Spectrosc. 243, 118662. doi: 10.1016/j.saa.2020.118662 32810775

[B146] Roberts C. A. Al-Tameemi H. M. Mashruwala A. A. Rosario-Cruz Z. Chauhan U. Sause W. E. . (2017). The Suf iron-sulfur cluster biosynthetic system is essential in Staphylococcus aureus, and decreased Suf function results in global metabolic defects and reduced survival in human neutrophils. Infect. Immun. 85, e00100-17. doi: 10.1128/IAI.00100-17 28320837 PMC5442634

[B147] Roier S. Adamo R. Rosini R. Pezzicoli A. Scarselli M. Petsch B. . (2025). An mRNA-based FimH nanoparticle vaccine against uropathogenic Escherichia coli is highly immunogenic in rodents. Front. Immunol. 16, 1668937. doi: 10.3389/fimmu.2025.1668937 41256840 PMC12620438

[B148] Rychlik I. Barrow P. A. (2005). *Salmonella* stress management and its relevance to behaviour during intestinal colonisation and infection. FEMS Microbiol. Rev. 29, 1021–1040. doi: 10.1016/j.femsre.2005.03.005 16023758

[B149] S C. Choi E. Cho Y. Nam D. Lee J. Lee E. (2019). The *Salmonella* virulence protein MgtC promotes phosphate uptake inside macrophages. Nat. Commun. 10, 3326. doi: 10.1038/s41467-019-11318-2 31346161 PMC6658541

[B150] Sabbagh S. C. Forest C. G. Lepage C. Leclerc J.-M. Daigle F. (2010). So similar, yet so different: Uncovering distinctive features in the genomes of *Salmonella enterica* serovars Typhimurium and Typhi: Genomic comparison of S. Typhimurium and S. Typhi. FEMS Microbiol. Lett. 305, 1–13. doi: 10.1111/j.1574-6968.2010.01904.x 20146749

[B151] Saha T. Arisoyin A. E. Bollu B. Ashok T. Babu A. Issani A. . (2023). Enteric fever: Diagnostic challenges and the importance of early intervention. Cureus 15, e41831. doi: 10.7759/cureus.41831 37575696 PMC10423039

[B152] Saha P. Xiao X. Yeoh B. S. Chen Q. Katkere B. Kirimanjeswara G. S. . (2018). The bacterial siderophore enterobactin confers survival advantage to *Salmonella* in macrophages. Gut Microbes 10, 412–423. doi: 10.1080/19490976.2018.1546519 30449241 PMC6546333

[B153] Serebnitskiy Z. Orban K. Finkel S. E. (2025). A role for Dps ferritin activity in long-term survival of Escherichia coli. Microbiol. Spectr. 13, e0183724. doi: 10.1128/spectrum.01837-24 40891860 PMC12502760

[B154] Sesardic D. Jones R. G. A. Leung T. Alsop T. Tierney R. (2004). Detection of antibodies against botulinum toxins. Movement Disorders: Off. J. Movement Disord. Soc. 19, S85–S91. doi: 10.1002/mds.20021 15027059

[B155] Sheikh A. Charles R. C. Rollins S. M. Harris J. B. Bhuiyan M. S. Khanam F. . (2010). Analysis of *Salmonella enterica* serotype Paratyphi A gene expression in the blood of bacteremic patients in Bangladesh. PloS Negl.Trop. Dis. 4, e908. doi: 10.1371/journal.pntd.0000908 21151879 PMC2998432

[B156] Siba V. Horwood P. F. Vanuga K. Wapling J. Sehuko R. Siba P. M. . (2012). Evaluation of serological diagnostic tests for typhoid fever in Papua New Guinea using a composite reference standard. Clin. Vaccine Immunol. 19, 1833–1837. doi: 10.1128/CVI.00380-12 22993409 PMC3491554

[B157] Sima C. M. Buzilă E. R. Trofin F. Păduraru D. Luncă C. Duhaniuc A. . (2024). Emerging strategies against non-typhoidal *Salmonella*: From pathogenesis to treatment. Curr. Issues Mol. Biol. 46, 7447–7472. doi: 10.3390/cimb46070442 39057083 PMC11275306

[B158] Singh S. P. Singh S. R. Williams Y. U. Jones L. Abdullah T. (1995). Antigenic determinants of the OmpC porin from *Salmonella* Typhimurium. Infect. Immun. 63, 4600–4605. doi: 10.1128/iai.63.12.4600-4605.1995 7591112 PMC173661

[B159] Sj L. Gebru A. E. Kim M. Park S. (2013). BaeR protein from *Salmonella enterica* serovar Paratyphi A induces inflammatory response in murine and human cell lines. Microbes Infection 15, 951–957. doi: 10.1016/j.micinf.2013.09.001 24055826

[B160] Smith E. W. Zhang X. Behzadi C. Andrews L. D. Cohen F. Chen Y. (2015). Structures of *Pseudomonas aeruginosa* LpxA reveal the basis for its substrate selectivity. Biochemistry 54, 5937–5948. doi: 10.1021/acs.biochem.5b00720 26352800

[B161] Snellings N. J. Johnson E. M. Baron L. S. (1977). Genetic basis of Vi antigen expression in *Salmonella Paratyphi* C. J. Bacteriol. 131, 57–62. doi: 10.1128/jb.131.1.57-62.1977 68953 PMC235390

[B162] Soldano A. Yao H. Punchi Hewage A. N. Meraz K. Annor-Gyamfi J. K. Bunce R. A. . (2021). Small molecule inhibitors of the bacterioferritin (BfrB)–ferredoxin (Bfd) complex kill biofilm-embedded *Pseudomonas aeruginosa* cells. ACS Infect. Dis. 7, 123–140. doi: 10.1021/acsinfecdis.0c00669 33269912 PMC7802073

[B163] Song J. Gao X. Galán J. E. (2013). Structure and function of the *Salmonella* Typhi chimaeric A(2)B(5) typhoid toxin. Nature 499, 350–354. doi: 10.1038/nature12377 23842500 PMC4144355

[B164] Spanò S. Ugalde J. E. Galán J. E. (2008). Delivery of a *Salmonella* Typhi exotoxin from a host intracellular compartment. Cell. Host Microbe 3, 30–38. doi: 10.1016/j.chom.2007.11.001 18191792

[B165] Srour S. Brown F. K. Sheffield J. W. ElGhazaly M. O’Connor D. Gibani M. M. . (2026). Typhoid toxin of *Salmonella* Typhi elicits host antimicrobial response during acute typhoid fever. EMBO Mol. Med. 18, 187–216. doi: 10.1038/s44321-025-00347-8 41326716 PMC12808722

[B166] Stepien T. A. Singletary L. A. Guerra F. E. Karlinsey J. E. Libby S. J. Jaslow S. L. . (2024). Nuclear factor kappa B-dependent persistence of *Salmonella* Typhi and Paratyphi in human macrophages. mBio 15, e0045424. doi: 10.1128/mbio.00454-24 38497655 PMC11005419

[B167] Szeto J. Namolovan A. Osborne S. E. Coombes B. K. Brumell J. H. (2009). *Salmonella*-containing vacuoles display centrifugal movement associated with cell-to-cell transfer in epithelial cells. Infect. Immun. 77, 996–1007. doi: 10.1128/IAI.01275-08 19103768 PMC2643626

[B168] Tan G. Cheng Z. Pang Y. Landry A. P. Li J. Lu J. . (2014). Copper binding in IscA inhibits iron‐sulphur cluster assembly in *E sch erichia coli*. Mol. Microbiol. 93, 629–644. doi: 10.1111/mmi.12676 24946160 PMC4132641

[B169] Tang S. W. Abubakar S. Devi S. Puthucheary S. Pang T. (1997). Induction and characterization of heat shock proteins of *Salmonella* Typhi and their reactivity with sera from patients with typhoid fever. Infect. Immun. 65, 2983–2986. doi: 10.1128/iai.65.7.2983-2986.1997 9199477 PMC175419

[B170] Thirumoorthy T. P. Bakthavatchalam Y. D. Jacob J. J. Manoharan Y. John J. Rupali P. . (2025). Azithromycin resistance in *Salmonella* Typhi: Navigating diagnostic uncertainties and clinical decision-making. JAC-Antimicrob. Resist. 7, dlaf107. doi: 10.1093/jacamr/dlaf107 40612528 PMC12219624

[B171] Townsend S. M. Kramer N. E. Edwards R. Baker S. Hamlin N. Simmonds M. . (2001). *Salmonella enterica* serovar Typhi possesses a unique repertoire of fimbrial gene sequences. Infect. Immun. 69, 2894–2901. doi: 10.1128/IAI.69.5.2894-2901.2001 11292704 PMC98240

[B172] Tran Vu Thieu N. Trinh Van T. Tran Tuan A. Klemm E. J. Ngoc Minh C. N. Voong Vinh P. . (2017). An evaluation of purified *Salmonella* Typhi protein antigens for the serological diagnosis of acute typhoid fever. J. Infection 75, 104–114. doi: 10.1016/j.jinf.2017.05.007 28551371 PMC5522525

[B173] Troxell B. Hassan H. M. (2013). Transcriptional regulation by ferric uptake regulator (Fur) in pathogenic bacteria. Front. Cell. Infect. Microbiol. 3. doi: 10.3389/fcimb.2013.00059 24106689 PMC3788343

[B174] Uchiya K. Kamimura Y. Jusakon A. Nikai T. (2019). *Salmonella* fimbrial protein FimH is involved in expression of proinflammatory cytokines in a toll-like receptor 4-dependent manner. Infect. Immun. 87, 10.1128/iai.00881–18. doi: 10.1128/iai.00881-18 30602501 PMC6386536

[B175] Upadhyay A. Pal D. Kumar A. (2024). Cellulase exhibited a therapeutic potential to inhibit *Salmonella enterica* serovar Typhi biofilm by targeting multiple regulatory proteins of biofilm. Microb. Pathogen. 196, 106979. doi: 10.1016/j.micpath.2024.106979 39326804

[B176] Urbina H. D. Silberg J. J. Hoff K. G. Vickery L. E. (2001). Transfer of sulfur from IscS to IscU during Fe/S cluster assembly. J. Biol. Chem. 276, 44521–44526. doi: 10.1074/jbc.M106907200 11577100

[B177] Uruén C. Chopo-Escuin G. Tommassen J. Mainar-Jaime R. C. Arenas J. (2020). Biofilms as promoters of bacterial antibiotic resistance and tolerance. Antibiotics 10, 3. doi: 10.3390/antibiotics10010003 33374551 PMC7822488

[B178] Verma S. K. (2009). Overexpression, purification, and immunogenicity of recombinant porin proteins of *Salmonella enterica* serovar Typhi (S. Typhi). J. Microbiol. Biotechnol. 19, 1034–1040. doi: 10.4014/jmb.0812.675 19809263

[B179] Vernikos G. S. Parkhill J. (2006). Interpolated variable order motifs for identification of horizontally acquired DNA: Revisiting the *Salmonella* pathogenicity islands. Bioinformatics 22, 2196–2203. doi: 10.1093/bioinformatics/btl369 16837528

[B180] Wagner C. Hensel M. (2011). Adhesive mechanisms of *Salmonella enterica*. Adv. Exp. Med. Biol. 715, 17–34. doi: 10.1007/978-94-007-0940-9_2 21557055

[B181] Wang B. X. Butler D. S. Hamblin M. Monack D. M. (2023). One species, different diseases: The unique molecular mechanisms that underlie the pathogenesis of typhoidal *Salmonella* infections. Curr. Opin. Microbiol. 72, 102262. doi: 10.1016/j.mib.2022.102262 36640585 PMC10023398

[B182] Wang M. Qazi I. H. Wang L. Zhou G. Han H. (2020). *Salmonella* virulence and immune escape. Microorganisms 8, 407. doi: 10.3390/microorganisms8030407 32183199 PMC7143636

[B183] Wangdi T. Winter S. E. Bäumler A. J. (2012). Typhoid fever: ‘You can’t hit what you can’t see.’. Gut Microbes 3, 88–92. doi: 10.4161/gmic.18602 22156762 PMC3370952

[B184] Wear S. S. Sande C. Ovchinnikova O. G. Preston A. Whitfield C. (2022). Investigation of core machinery for biosynthesis of Vi antigen capsular polysaccharides in gram-negative bacteria. J. Biol. Chem. 298, 101486. doi: 10.1016/j.jbc.2021.101486 34896394 PMC8760489

[B185] Weckener M. Woodward L. S. Clarke B. R. Liu H. Ward P. N. Le Bas A. . (2023). The lipid linked oligosaccharide polymerase Wzy and its regulating co-polymerase, Wzz, from enterobacterial common antigen biosynthesis form a complex. Open Biol. 13, 220373. doi: 10.1098/rsob.220373 36944376 PMC10030265

[B186] Wellawa D. H. Lam P.-K. S. White A. P. Gomis S. Allan B. Köster W. (2022). High affinity iron acquisition systems facilitate but are not essential for colonization of chickens by *Salmonella* Enteritidis. Front. Microbiol. 13. doi: 10.3389/fmicb.2022.824052 35308377 PMC8928163

[B187] Wen Y. Ouyang Z. Devreese B. He W. Shao Y. Lu W. . (2017). Crystal structure of master biofilm regulator CsgD regulatory domain reveals an atypical receiver domain. Protein Sci. 26, 2073–2082. doi: 10.1002/pro.3245 28758290 PMC5606546

[B188] Wetter M. Goulding D. Pickard D. Kowarik M. Waechter C. J. Dougan G. . (2012). Molecular characterization of the viaB locus encoding the biosynthetic machinery for Vi capsule formation in *Salmonella* Typhi. PloS One 7, e45609. doi: 10.1371/journal.pone.0045609 23029132 PMC3448643

[B189] Whittington D. A. Rusche K. M. Shin H. Fierke C. A. Christianson D. W. (2003). Crystal structure of LpxC, a zinc-dependent deacetylase essential for endotoxin biosynthesis. Proc. Natl. Acad. Sci. 100, 8146–8150. doi: 10.1073/pnas.1432990100 12819349 PMC166197

[B190] Wilson R. P. Winter S. E. Spees A. M. Winter M. G. Nishimori J. H. Sanchez J. F. . (2011). The Vi capsular polysaccharide prevents complement receptor 3-mediated clearance of *Salmonella enterica* serotype Typhi. Infect. Immun. 79, 830–837. doi: 10.1128/IAI.00961-10 21098104 PMC3028862

[B191] Winter S. E. Winter M. G. Atluri V. Poon V. Romão E. L. Tsolis R. M. . (2015). The flagellar regulator TviA reduces pyroptosis by *Salmonella enterica* serovar Typhi. Infect. Immun. 83, 1546–1555. doi: 10.1128/IAI.02803-14 25644011 PMC4363433

[B192] Winter S. E. Winter M. G. Thiennimitr P. Gerriets V. A. Nuccio S.-P. Rüssmann H. . (2009). The TviA auxiliary protein renders the *Salmonella enterica* serotype Typhi RcsB regulon responsive to changes in osmolarity. Mol. Microbiol. 74, 175–193. doi: 10.1111/j.1365-2958.2009.06859.x 19703107 PMC2763492

[B193] Wizemann T. M. Adamou J. E. Langermann S. (1999). Adhesins as targets for vaccine development. Emerging Infect. Dis. 5, 395–403. doi: 10.3201/eid0503.990310 10341176 PMC2640765

[B194] Xie L. Ming L. Ding M. Deng L. Liu M. Cong Y. (2022). Paratyphoid fever A: Infection and prevention. Front. Microbiol. 13. doi: 10.3389/fmicb.2022.945235 35875577 PMC9304857

[B195] Yao H. Soldano A. Fontenot L. Donnarumma F. Lovell S. Chandler J. R. . (2022). Pseudomonas aeruginosa bacterioferritin is assembled from FtnA and BfrB subunits with the relative proportions dependent on the environmental oxygen availability. Biomolecules 12, 366. doi: 10.3390/biom12030366 35327558 PMC8945002

[B196] Young H. E. Donohue M. P. Smirnova T. I. Smirnov A. I. Zhou P. (2013). The UDP-diacylglucosamine pyrophosphohydrolase LpxH in lipid A biosynthesis utilizes Mn2+ cluster for catalysis. J. Biol. Chem. 288, 26987–27001. doi: 10.1074/jbc.M113.497636 23897835 PMC3779701

[B197] Zhang H. Zhou Y. Bao H. Liu H. (2006). Vi antigen biosynthesis in *Salmonella Typhi* : Characterization of UDP- *N* -acetylglucosamine C-6 dehydrogenase (TviB) and UDP- *N* -acetylglucosaminuronic acid C-4 epimerase (TviC). Biochemistry 45, 8163–8173. doi: 10.1021/bi060446d 16800641 PMC2515272

[B198] Zhao H. Zhang X. Zhang N. Zhu L. Lian H. (2025). The interplay between *Salmonella* and host: Mechanisms and strategies for bacterial survival. Cell. Insight 4, 100237. doi: 10.1016/j.cellin.2025.100237 40177681 PMC11964643

[B199] Zhou P. Zhao J. (2017). Structure, inhibition, and regulation of essential lipid A enzymes. Biochim. Biophys. Acta 1862, 1424–1438. doi: 10.1016/j.bbalip.2016.11.014 27940308 PMC5466501

[B200] Zhu C. Xiong K. Chen Z. Hu X. Li J. Wang Y. . (2015). Construction of an attenuated *Salmonella enterica* serovar Paratyphi A vaccine strain harboring defined mutations in htrA and yncD. Microbiol. Immunol. 59, 443–451. doi: 10.1111/1348-0421.12276 26084199

